# Supplementary material for: Single-cell intracellular epitope and transcript detection reveals signal transduction dynamics
Source: Cell Rep Methods. 2021 Sep 15;1(5):100070. doi: 10.1016/j.crmeth.2021.100070 (PMC9017125; doi:10.1016/j.crmeth.2021.100070)
Supplement: Document S1. Figures S1–S5 and Tables S1–S3 [file mmc1.pdf]

Cell Reports Methods, Volume *1*

Supplemental information

Single-cell intracellular epitope and transcript detection reveals signal transduction dynamics

Francesca Rivello, Erik van Buijtenen, Kinga Matuła, Jessie A.G.L. van Buggenum, Paul Vink, Hans van Eenennaam, Klaas W. Mulder, and Wilhelm T.S. Huck

Supplementary Information

Supplementary Figures

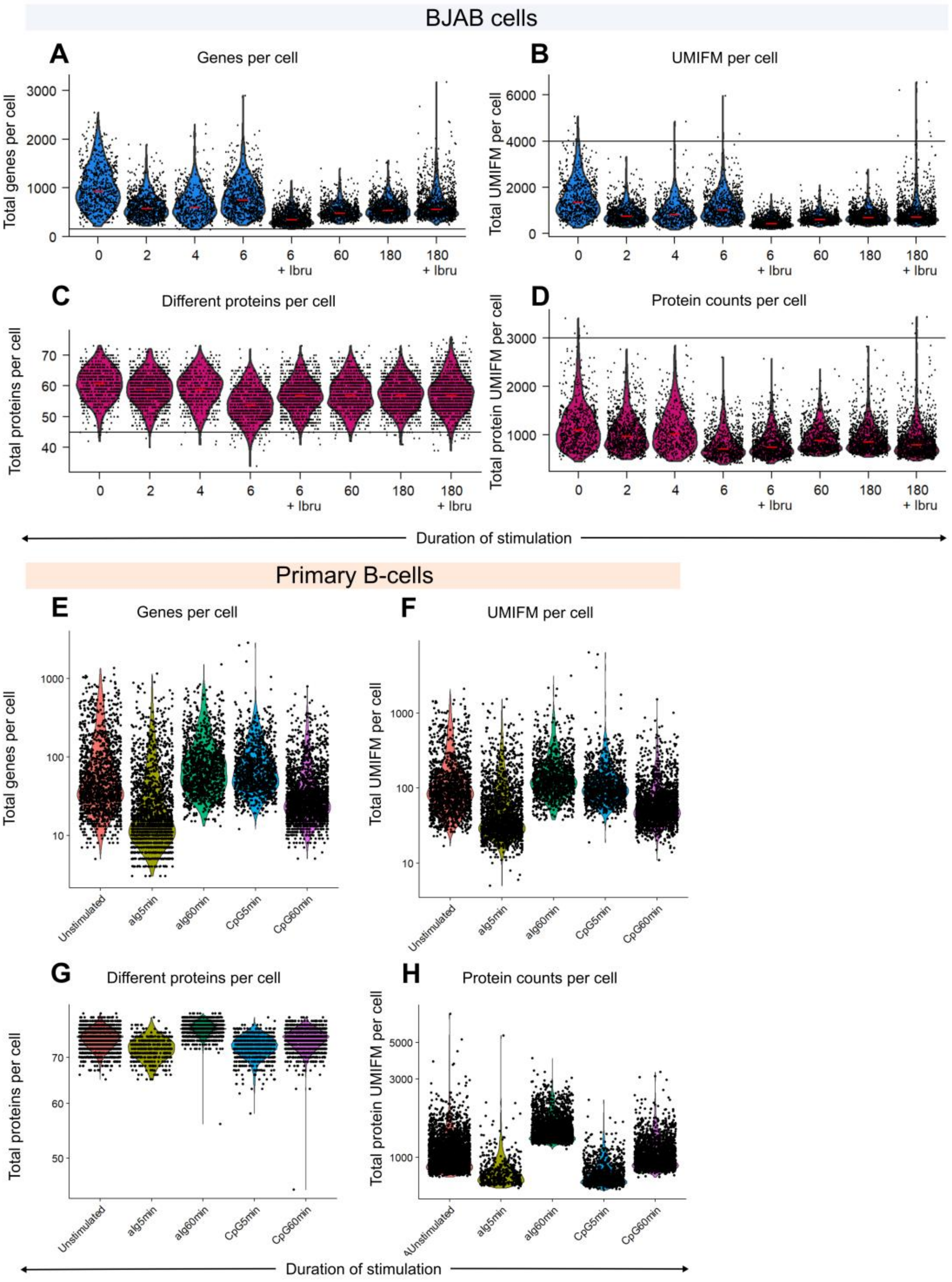

Supplementary Figure 1: Technical quality of the QuRIE-seq transcriptomic and proteomic libraries on BJAB cells (A-D) and primary B-cells isolated from PBMCs (E-H). (**A-D**) BJAB cells at eleven different stimulation and/or inhibition conditions: after stimulation with aIg for: 0, 2, 4, 6, 60 and 180 minutes, with or without prior Ibrutinib inhibition. (**E-H**) Primary B-cells isolated from PBMCs after stimulation with aIg or CpG for: 0, 5 and 60 minutes. Violin plots for (**A, B, E, F**) the transcriptomic and (**C, D, G, H**) the proteomic libraries. (**A**, **E**) The number of different genes detected per cell. (**B, F**) The number of unique molecular identifiers filtered mapped (UMIFM) detected per cell. (**C, G**) The number of different proteins detected per cell. (**D, H**) The number of protein counts per cell.

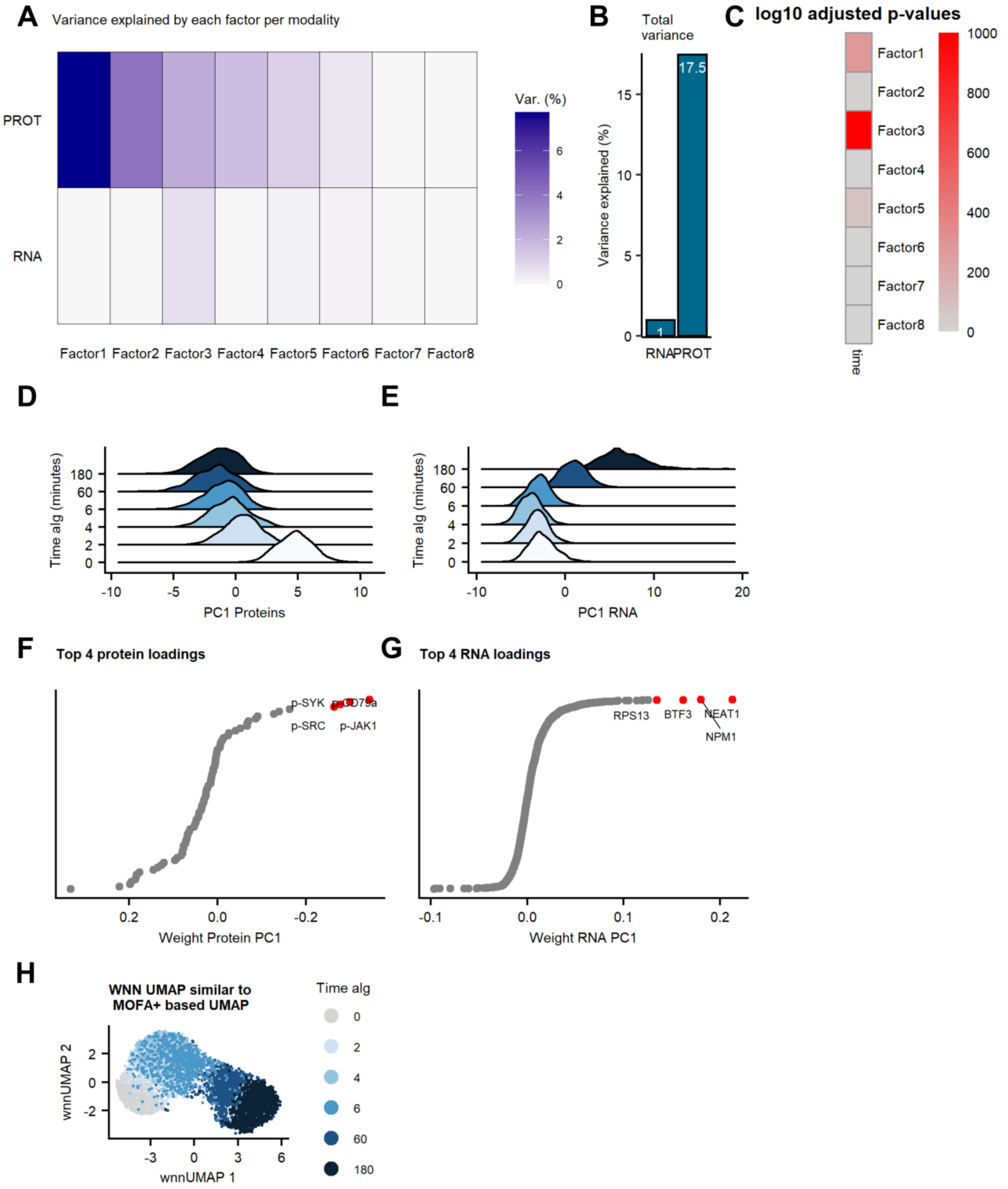

Supplementary Figure 2: Supplementary results on MOFA+ analysis related to Figure 2. (**A**) Percentage of variance explained by each factor per modality (proteins and RNA). (**B**) Percentage of the total variance explained per modality. (**C**) Pearson's correlation (p-value) between the MOFA+ factors and the duration of anti-immunoglobulin antibody (aIg) stimulation. (**D-H**) Principal component analysis (PCA) and (Weighted Nearest Neighbor Analysis) WNN analysis using the Seurat package shows similar results to

factor 1 and factor 3 determined by MOFA+ analysis. (**D**) Density plot of protein-based PC1 values as the function of time after stimulation with aIg. (**E**) Density plot of RNA-based PC1 values as function of time after stimulation with aIg. (**F**) Protein loadings contributing to PC1. The top 4 protein loadings are annotated. (**G**) RNA loadings contributing to PC1. The top 4 RNA loadings are annotated. The results are equivalent to factors 1 and 3 in MOFA+ analysis, respectively. (**H**) UMAP of weighted nearest neighbors computed from RNA and Protein PC's (first 7 dimensions) using Seurat V4.

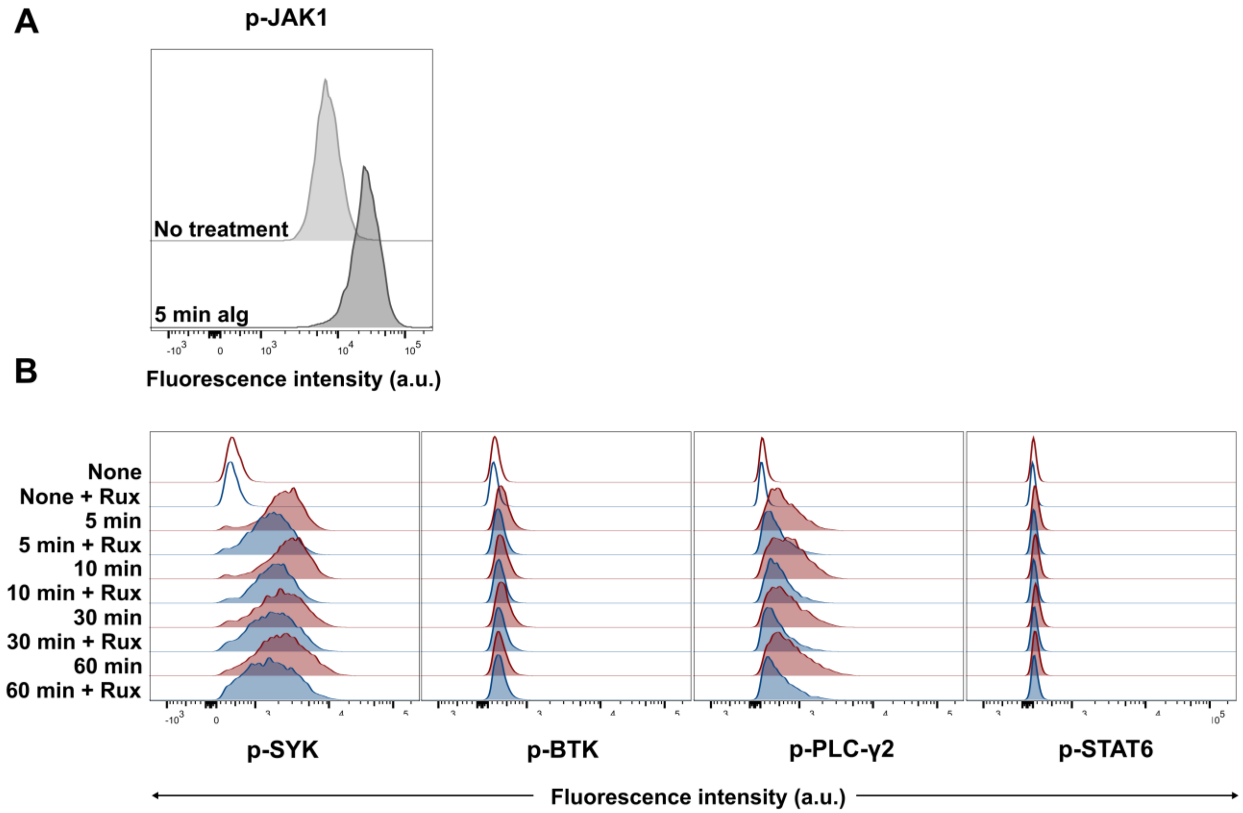

Supplementary Figure 3: The role of JAK1 in the BCR response to stimulation with aIg. Related to Figure 2. (**A**) JAK1 phosphorylation after 5 minutes of stimulation with aIg characterized by flow cytometry. (**B**) The phosphorylation level of four proteins: SYK, BTK, PLC-γ2, and STAT6 determined using flow cytometry for unstimulated BJAB cells, and stimulated with aIg for: 5, 10, 30, and 60 minutes with (blue curves) or without (red curves) prior Ruxolitinib (Rux) inhibition. *Rux is a JAK1/2 inhibitor.*

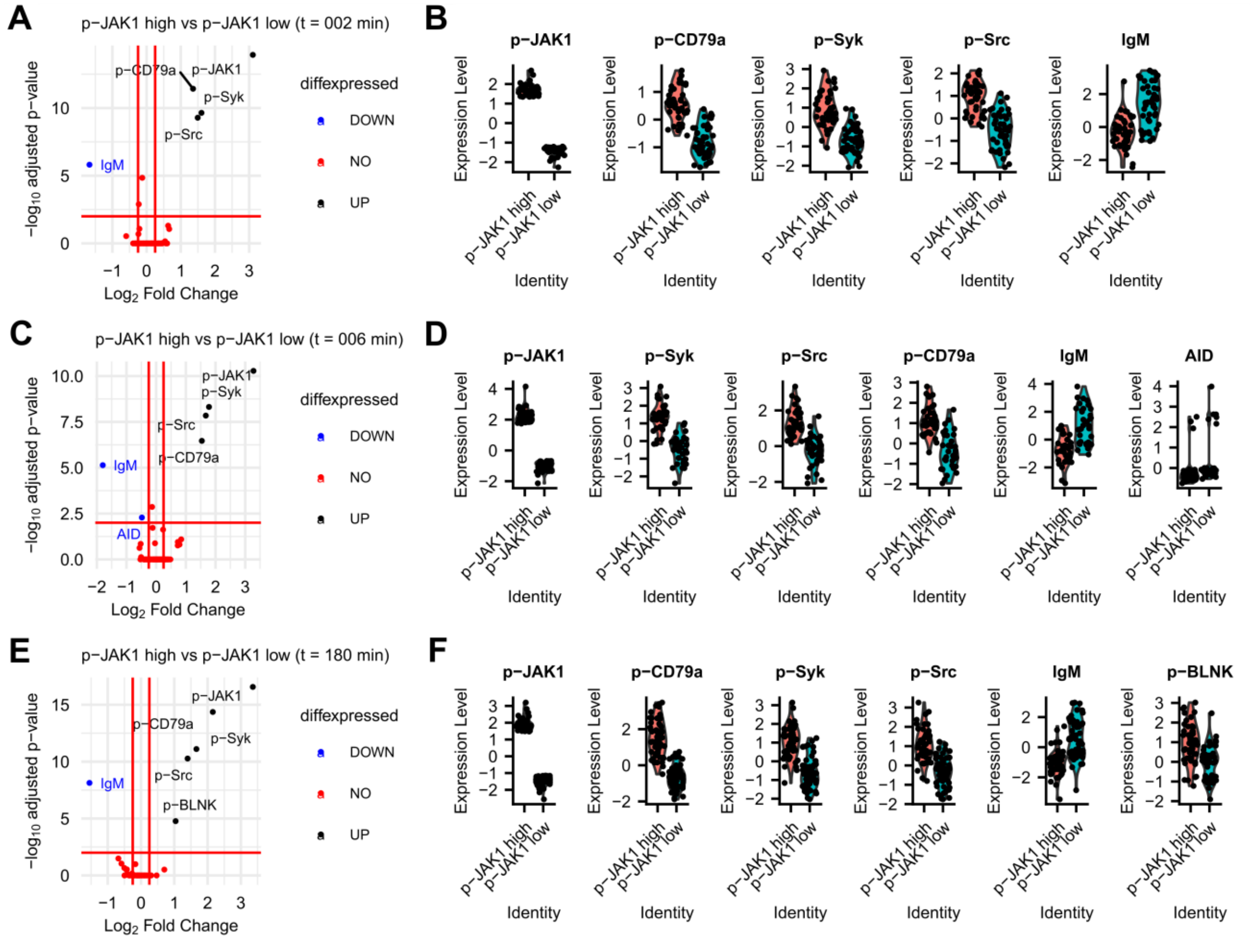

Supplementary Figure 4. Differential (phospho-)proteins of top 5% versus bottom 5% p-JAK1 cells. At timepoint t = 2 min (**A, B**), t = 6 min (**C, D**) or t = 180 min (**E, F**) differential expression analysis was performed on normalized and scaled counts, between the subset of cells with high (top 5%) p-JAK1 signal versus low (bottom 5%) p-JAK1 signal (Wilcoxon Rank Sum test). Volcano plots (**A, C, E**) show significant increased or decreased phospho-proteins (Log_2 Fold Change > 0.25 or < -0.25; adjusted p-val < 0.05). Violin plots (**B, D, F**) illustrate the highlighted significant features.

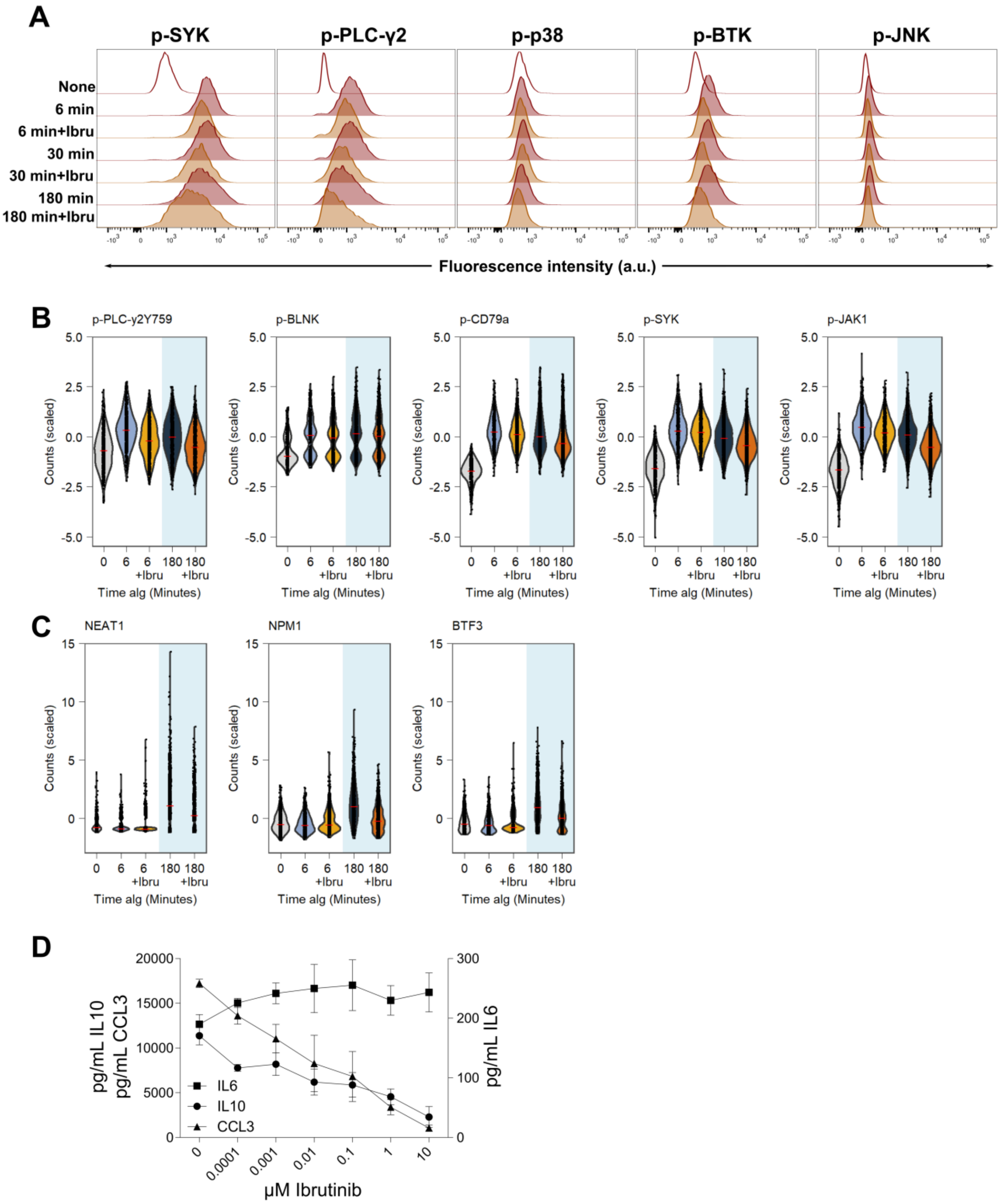

Supplementary Figure 5: Cellular response to anti-immunoglobulin antibody (aIg) stimulation in the presence of Ibrutinib (Ibru) inhibitor of Bruton's tyrosine kinase (BTK) . Related to Figure 3. (**A**) Flow cytometry characterization of the phosphorylation of SYK, PLC-γ2, p38, BTK, and JNK upon different stimulation conditions: unstimulated cells (0 minutes - red empty line); stimulation with aIg for: 6, 30, and 180 minutes with (orange filled line) and without (red filled line) prior Ibru inhibition. (**B**) QuRIE-seq

measurement of phosphoproteins. Scaled counts of signaling components up- or downstream of BTK: PLC-y2, BLNK, CD79a, SYK, and JAK1 as a function of stimulation with aIg for: 0, 6, and 180 minutes with and without Ibru. (**C**) Top 3 genes of factor 3 loadings in the QuRIE-seq measurement: *NEAT1*, *NPM1*, and *BTF3*, scaled counts as a function of stimulation with aIg for: 0, 6, and 180 minutes with and without Ibru. (**D**) ELISA analysis of BJABs culture supernatant for cytokines upon overnight (16 hours) stimulation in the presence of Ibrutinib. Ibrutinib was used to block BTK (part of BCR signaling pathway), and titrated while cells were stimulated with aIg (final concentration of 10 μg/ml). The dose-dependent decrease of cytokine secretion was observed for IL10 and CCL3, but not for IL6.

Supplementary Tables

Supplementary Table 1: Antibodies used for immunostaining of cells prior to microfluidic encapsulation. Related to STAR Methods section.

	Target	Ab barcode sequence	Vendor	Clone	Used concentration
1	**Histone H3**	**CAATCCCT**	Cell Signaling 4499BF	D1H2	0.1 µg/ml
2	**p-p65**	**GTCCAGGC**	Cell Signaling 3033BF	93H1	1 µg/ml
3	**p-SYK**	**TGTGTATA**	Cell Signaling 2710BF	C87C1	1 µg/ml
4	**p-JNK**	**AGGATCGA**	Cell Signaling 9255BF	G9	1 µg/ml
5	**p-p38**	**CACGATTC**	Cell Signaling 4511BF	D3F9	1 µg/ml
6	**p-PLC-y2**	**GTATCGAG**	R&D Systems MAB37161	790623	1 µg/ml
7	**p-BTK**	**TCTCGACT**	Biolegend 601702	A16128B	1 µg/ml
8	**p-SHP-2**	**ACCCGCAC**	Cell Signaling 5431BFF	D66F10	1 µg/ml
9	**p-SHP-1**	**CATGCGTA**	Cell Signaling 8849BF	D11G5	1 µg/ml
10	**Cyclin E**	**GTGATAGT**	ThermoFisher 32-1600	HE12	1 µg/ml
11	**c-MYC**	**TGATATCG**	ThermoFisher 700648	27H46L35	1 µg/ml
12	**p-RB**	**AGGGCGTT**	ThermoFisher 701059	14H7L14	0.1 µg/ml
13	**p-c-JUN**	**CTATACGC**	ThermoFisher MA5-27760	GT653)	1 µg/ml
14	**active Caspase-3**	**GCTCGTCA**	BD Bioscience 559565	C92-605	1 µg/ml
15	**CD70**	**TACATAAG**	BD Bioscience 555833	Ki-24	1 µg/ml
16	**IgD**	**AATTGAAC**	BD Bioscience 555776	IA6-2	1 µg/ml
17	**CD86**	**CCAGTGGA**	BD Bioscience 555655	2331 (FUN-1)	1 µg/ml
18	**CD20**	**GTCCATTG**	BD Bioscience 555677	H1	0.1 µg/ml
19	**CD79a**	**TGGACCCT**	BD Bioscience 555934	HM47	0.1 µg/ml
20	**CD5**	**AGCAGTTA**	BD Bioscience 555350	UCHT2	1 µg/ml
21	**p-AKT**	**CTTGTACC**	Cell Signaling 4060BF	D9E	1 µg/ml
22	**S6**	**GAACCCGG**	Cell Signaling 2317B	54D2	1 µg/ml
23	**ERK 1/2**	**TCGTAGAT**	Cell Signaling 4696BF	L34F12	1 µg/ml
24	**p38**	**ACGCGGAA**	Cell Signaling 8690BF	D13E1	1 µg/ml
25	**AKT**	**CGCTATCC**	Cell Signaling 4685BF	11EE7	1 µg/ml
26	**SYK**	**GTTGCATG**	Cell Signaling 13198BF	D3Z1E	1 µg/ml
27	**BTK**	**TAAATCGT**	Cell Signaling 8547BF	D3H5	0.1 µg/ml
28	**p-S6**	**ATCGCCAT**	Cell Signaling 4858BF	D57.2.2E	0.1 µg/ml
29	**p-ERK 1/2**	**CATAAAGG**	Cell Signaling 5726BF	D1H6G	1 µg/ml
30	**p65**	**TCACGGTA**	Cell Signaling 8242BF	D14E12	0.1 µg/ml
31	**CD19**	**CACTCAAC**	SantaCruz sc-373897	F-3	2 µg/ml
32	**p-Histon H3**	**GCTGTGA**	Biolegend 650802	11D8	0.1 µg/ml
33	**IgM**	**TTGCGTCG**	Biolegend 314502	MHM-88	1 µg/ml
34	**Cyclin A**	**ATATGAGA**	Biolegend 644001	E23.1	0.1 µg/ml
35	**p-Histon H2A.X**	**CACCTCAG**	Biolegend 613402	2F3	0.1 µg/ml

**36**	**Cyclin B1**	**GCTACTTC**	Biolegend 647902	V152	0.1 µg/ml
**37**	**Ki-67**	**TGGGAGCT**	Biolegend 350523	Ki-67	0.1 µg/ml
**38**	**JNK**	**ATCCGGCA**	R&D Systems AF1387		1 µg/ml
**39**	**SHP-1**	**CCGTTATG**	R&D Systems MAB1878	255402	1 µg/ml
**40**	**p-SRC**	**GGTAATGT**	R&D Systems MAB2685	1246F	1 µg/ml
**41**	**p-TOR**	**TAAGCCAC**	R&D Systems MAB1665	834115	0.1 µg/ml
**42**	**GAPDH**	**ACCGAACA**	Biolegend 607902	W17079A	1 µg/ml
**43**	**BLNK**	**GTTTGTGG**	BD Biosciences 559930	2B11	1 µg/ml
**44**	**p-BLNK**	**TAGACGAC**	BD Biosciences 558366	J117-1278	0.1 µg/ml
**45**	**p-PKC-b1**	**ACGCTTGG**	ThermoFisher 702430	3H8L1	0.1 µg/ml
**46**	**CD53**	**CGCTACAT**	BD Biosciences 555506	HI29	0.1 µg/ml
**47**	**CD38**	**GAAAGACA**	Biolegend 303535	HIT2	0.1 µg/ml
**48**	**CD45**	**TTTGCGTC**	Biolegend 304045	HI30	0.1 µg/ml
**49**	**p-IRAK4**	**ATGGTCGC**	Cell Signaling 11927S	D6D7	1 µg/ml
**50**	**p-CD79a**	**CGACATAG**	Cell Signaling 14732BF	D1B9	0.1 µg/ml
**51**	**p-CDK1**	**GATTCGCT**	ThermoFisher 701808	17H29L7	1 µg/ml
**52**	**p-CDK4**	**TCCAGATA**	ThermoFisher 702556	9H2L7	1 µg/ml
**53**	**p-AMPK-a1/2**	**ACTACTGT**	ThermoFisher701068	10H2L20	0.1 µg/ml
**54**	**p-AMPK-b1**	**CGGGAACG**	Thermo Fisher 700241	9H26L42	0.1 µg/ml
**55**	**T-bet**	**GACCTCTC**	Biolegend 644825	4B10	1 µg/ml
**56**	**p-IKK a/b**	**TTATGGAA**	ThermoFisher 701643	7H17L17	1 µg/ml
**57**	**CD27**	**ACAGCAAC**	Abcam ab192336	EPR8569	0.1 µg/ml
**58**	**p-JAK1**	**CGCAATTT**	ThermoFisher 700028	59H4L5	1 µg/ml
**59**	**p-PLC-y2 (Y759)**	**GAGTTGCG**	R&D Systems MAB7377	744757	0.1 µg/ml
**60**	**IL10**	**TTTCGCGA**	ThermoFisher 16-7108-85	JES3-9D7	1 µg/ml
**61**	**CCL3/4**	**ACCAGTCC**	R&D Systems MAB2701-100	93342	1 µg/ml
**62**	**CD80**	**CTTTCCTT**	Biolegend 305212	2D10	1 µg/ml
**63**	**p-CDK6**	**CTGGACGT**	ThermoFisher	16HCLC	1 µg/ml
**64**	**p-STAT1**	**GAACGGTC**	ThermoFisher 33-3400	ST1P-11A5	1 µg/ml
**65**	**p-STAT3**	**TGTTCACG**	Biolegend 690402	A16002B	1 µg/ml
**66**	**p-STAT5**	**ATCTGATC**	ThermoFisher 701063	6H5L15	1 µg/ml
**67**	**p-STAT6**	**GAGAAGGG**	ThermoFisher 700247	46H1L12	1 µg/ml
**68**	**KLF6**	**TCACTCCT**	ThermoFisher 39-6900	9A2	1 µg/ml
**69**	**BCL6**	**AGATAACA**	BD Bioscneces 561520	K112-91	1 µg/ml
**70**	**AID**	**CTTATTTG**	BD Biosciences 565784	EK2-5G9	1 µg/ml
**71**	**IgG**	**GCGGGCAT**	BD Biosciences 555784	G18-145	0.1 µg/ml
**72**	**CD24**	**TACCCGGC**	RnD Systems MAB5247	ML5	1 µg/ml
**73**	**IgA**	**ATTGTTTC**	Biolegend 411502	HP6123	0.5 µg/ml
**74**	**IgE**	**CGCAGGAG**	Biolegend 325502	MHE-18	1 µg/ml
**75**	**CD23**	**GCACCAGT**	Abcam ab245732	SP163	0.1 µg/ml

**76**	**BLIMP1**	**TAGTACCA**	RnD Systems MAB36081	646702	1 µg/ml
**77**	**IRF8**	**ATTCGTGC**	Biolegend 656502	656502	1 µg/ml
**78**	**IRF4**	**CGCGTGCA**	ThermoFisher 14-9858-82	3 E 4	1 µg/ml
**79**	**BAFF-R**	**GAATACTG**	RnD Systems MAB1162	2403C	1 µg/ml
**80**	**XBP1**	**TCGACAAT**	Abcam ab239954	EPR4086	1 µg/ml

Supplementary Table 2: Antibodies used for flow cytometry analysis of BJABs. Related to STAR Methods section.

Target	Fluorophore	Vendor	Clone	Used concentration
**p-SYK**	PE	Cell Signaling 6485	C87C1	1/50
**p-PLC-γ2**	Alexa 647	BD Bioscience 558498	K86-689.37	1/100
**p-BTK**	PE	Biolegend 601704	A16128B	1/50
**p-p38**	Alexa 488	Cell Signaling 41768S	3D7	1/50
**p-AKT**	PE	Cell Signaling 5315	D9E	1/50
**p-ERK 1/2**	PE	Cell Signaling 75765S	D1H6G	1/50
**p-JNK**	Alexa 647	Cell Signaling 9257S	G9	1/50
**p-p65**	Alexa 647	Cell Signaling 5733	93H1	1/50
**p-S6**	PE	Cell Signaling 5316	D57.2.2E	1/800

Supplementary Table 3: Sequences of all the primers used for library preparation. Related to STAR Methods section. All primers were ordered from Biolegio (The Netherlands).

	Primer sequence 5' -> 3'	Length (bp)
**PE2-N6**	TCGGCATTCCTGCTGAACCGCTCTTCCGATCT NNNNN	38
**PE1**	CAAGCAGAAGACGGCATACGAGAT [6-bp library index] CTCTTTCCCTACACGA	46
**PE2**	AATGATACGGCGACCACCGAGATCTACACGGTCTCGGCATTCCTGCTG AAC	51
**Custom Read 1 primer**	GGCATTCCTGCTGAACCGCTCTTCCGATCT	30
**Custom Index Read primer**	AGATCGGAAGAGCGTCGTGTAGGGAAAGAG	30
**Custom Read 2 primer**	CTCTTTCCCTACACGACGCTCTTCCGATCT	30
