# Supplementary material for: Single-cell intracellular epitope and transcript detection reveals signal transduction dynamics
Source: Cell Rep Methods. 2021 Sep 15;1(5):100070. doi: 10.1016/j.crmeth.2021.100070 (PMC9017125; doi:10.1016/j.crmeth.2021.100070)
Supplement: Document S2. Article plus supplemental information [file mmc2.pdf]

Cell Reports
Methods

Report

Single-cell intracellular epitope and transcript detection reveals signal transduction dynamics

Graphical abstract

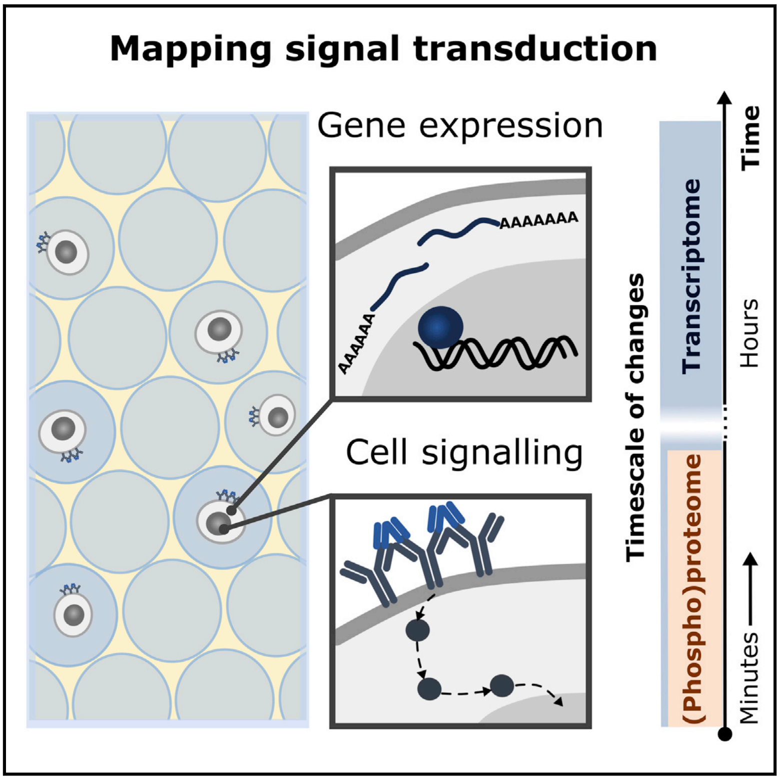

Highlights

- QuRIE-seq quantifies single-cell mRNA and intra- and extracellular (phospho)proteins
- Multi-modal analysis shows changes in protein and mRNA levels at different timescales
- JAK1 is shown to be involved in BCR signaling

Authors

Francesca Rivello, Erik van Buijtenen, Kinga Matuła, ..., Hans van Eenennaam, Klaas W. Mulder, Wilhelm T.S. Huck

Correspondence

k.mulder@science.ru.nl (K.W.M.),
w.huck@science.ru.nl (W.T.S.H.)

In brief

Rivello et al. develop QuRIE-seq for high-throughput quantification of single-cell mRNA levels in combination with extra- and intracellular protein levels via immunostaining with 80 DNA-conjugated antibodies. This method shows the cellular response over time to external perturbations at the surface marker, (phospho)proteome, or gene expression level.

Rivello et al., 2021, Cell Reports Methods 1, 100070
September 27, 2021 © 2021 The Author(s).
https://doi.org/10.1016/j.crmeth.2021.100070

CellPress

Cell Reports Methods

CellPress
OPEN ACCESS

Report

Single-cell intracellular epitope and transcript detection reveals signal transduction dynamics

Francesca Rivello,[1,4] Erik van Buijtenen,[1,4] Kinga Matuła,[1,4] Jessie A.G.L. van Buggenum,[1,4] Paul Vink,[2]
Hans van Eenennaam,[2] Klaas W. Mulder,[3,*] and Wilhelm T.S. Huck[1,5,*]
[1]Institute for Molecules and Materials, Radboud University, Nijmegen 6525 AJ, the Netherlands
[2]Aduro Biotech, Oss 5349 AB, the Netherlands
[3]Radboud Institute for Molecular Life Sciences, Radboud University, Nijmegen 6525 GA, the Netherlands
[4]These authors contributed equally
[5]Lead contact
*Correspondence: k.mulder@science.ru.nl (K.W.M.), w.huck@science.ru.nl (W.T.S.H.)
https://doi.org/10.1016/j.crmeth.2021.100070

MOTIVATION External stimuli lead to a range of dynamic intracellular responses. There is a dearth of methods that simultaneously capture the dynamics of this response at the levels of signaling and gene expression networks. We combined high-throughput single-cell RNA sequencing with intracellular epitope detection via DNA-barcoded antibodies, thus opening a window on the dynamics of signal transduction.

SUMMARY

To further our understanding of how biochemical information flows through cells upon external stimulation, we require single-cell multi-omics methods that concurrently map changes in (phospho)protein levels across signaling networks and the associated gene expression profiles. Here, we present quantification of RNA and intracellular epitopes by sequencing (QuRIE-seq), a droplet-based platform for single-cell RNA and intra- and extracellular (phospho)protein quantification through sequencing. We applied QuRIE-seq to quantify cell-state changes at both the signaling and the transcriptome level after 2-, 4-, 6-, 60-, and 180-min stimulation of the B cell receptor pathway in Burkitt lymphoma cells. Using the multi-omics factor analysis (MOFA+) framework, we delineated changes in single-cell (phospho)protein and gene expression patterns over multiple timescales and revealed the effect of an inhibitory drug (ibrutinib) on signaling and gene expression landscapes.

INTRODUCTION

The processing of information from the outside environment through signaling pathways is one of the most fundamental processes determining cellular phenotype, function, and fate. Single-cell multi-modal omics tools are changing our understanding of biology, but we still lack high-throughput single-cell techniques that capture both changes in phosphorylation levels and the much slower ensuing changes in gene expression patterns (Zhu et al., 2020). Targeted methods like REAP-seq (Peterson et al., 2017), CITE-seq (Stoeckius et al., 2017), or ECCITE-seq (Mimitou et al., 2019) measure simultaneously transcripts and proteins in single cells by using DNA-tagged antibodies, but are limited to the detection of surface epitopes. Our recent work showed a strategy for plate-based single-cell transcriptome and intracellular protein measurements for six epitopes (Gerlach et al., 2019). Here, we present quantification of RNA and intracellular epitopes by sequencing (QuRIE-seq), a high-throughput droplet-based platform to simultaneously quantify 80 intra- and extracellular (phospho)proteins and the transcriptome from thousands of individual cells.

RESULTS

QuRIE-seq workflow

We developed QuRIE-seq to study signal transduction and downstream transcriptional changes by using a validated panel of DNA-barcoded antibodies targeting components of the B lymphocyte (B cell) antigen receptor (BCR) signaling pathway, as well as cell-cycle and surface markers (Table S1). To allow intracellular epitope detection, we cross-linked cells by using a mix of reversible fixatives (dithiobis(succinimidyl propionate)/succinimidyl 3-(2-pyridyldithio)propionate [DSP/SPDP]), permeabilized, stained with the DNA-barcoded antibody panel, and co-compartmentalized in nanoliter droplets with single-cell barcoded primer-loaded gel beads (based on the inDrop protocol) (Klein et al., 2015; Zilionis et al., 2017), before further library preparation and sequencing (Figures 1A–1E). Reversible fixation

This is an open access article under the CC BY license (http://creativecommons.org/licenses/by/4.0/).

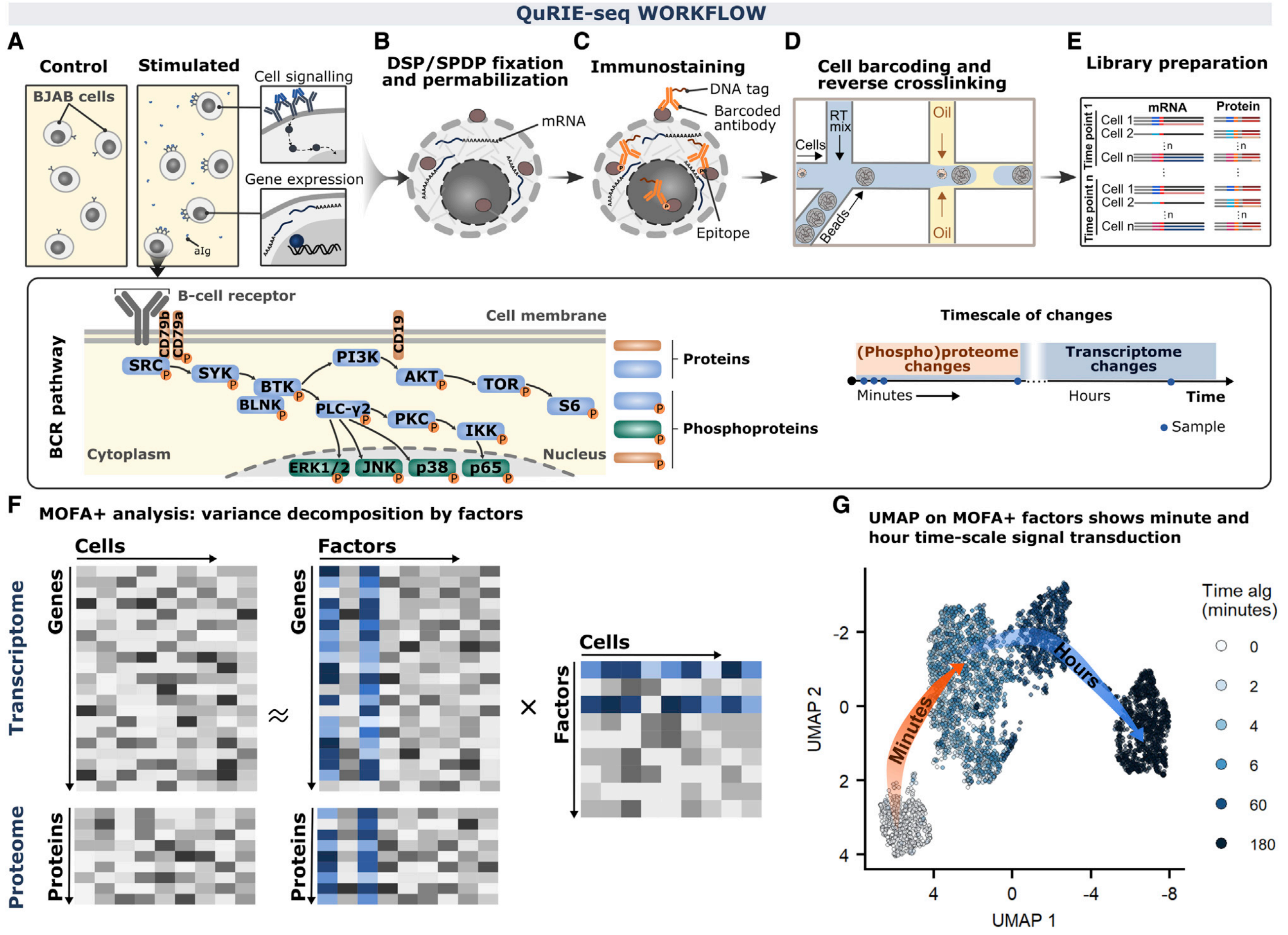

Figure 1. Multi-modal signal transduction in BJAB cells at the single-cell resolution by using quantification of RNA and intracellular epitopes (QuRIE-seq) combined with multi-omics factor analysis (MOFA+)

(A–G) Schematic overview of the QuRIE-seq workflow for combined single-cell RNA sequencing and (phospho)protein measurement.

(A) The dynamic response of BJAB cells to stimulation for different durations with polyclonal anti-immunoglobulin antibody (aIg). The scheme of part of the targeted proteins in the B cell antigen receptor (BCR) signaling pathway is shown. The timescale of (phospho)proteome changes falls into a minute regime, whereas most significant transcriptome changes are observed after hours.

(B) Cells are reversibly cross-linked with DSP/SPDP (fixatives) and permeabilized.

(C) DNA-tagged antibodies targeting membrane and intracellular (phospho)proteins are used for immunostaining.

(D) Single cells are co-encapsulated in a high-throughput manner in nanoliter droplets with barcoding hydrogel beads.

(E) Preparation of two libraries (proteome and transcriptome) is followed by sequencing.

(F) Data analysis overview: MOFA+ takes sequencing data matrices from both modalities (transcriptome and proteome) and decomposes these into matrices containing factors that can be analyzed further downstream.

(G) UMAP embedding based on the MOFA+ factors (factor 1 to 7) for 4,754 BJAB cells stimulated with aIg for different durations: 0, 2, 4, 6, 60, and 180 min.

of Burkitt lymphoma (BJAB) cells with DSP/SPDP and permeabilization showed similar or higher (phospho)protein signals and signal-to-noise ratios by flow cytometry and were equivalent to conventional paraformaldehyde fixation methods. For this BJAB cancer cell line, the fixation and permeabilization method resulted in a gene-detection rate comparable to that of unfixed single-cell analysis, similar to previously published results in primary keratinocytes (Gerlach et al., 2019). Technical quality checks of the transcriptomic and proteomic data generated with QuRIE-seq for fixed BJAB cells are presented in Figures S1A–S1D. Analysis of primary B cells isolated from peripheral blood mononuclear cells resulted in a good protein library (high protein counts and apparent detection of >70 proteins), but low-quality RNA library (~100 genes/cell and high percentage of mitochondrial counts), prompting us not to analyze this data-set further (Figures S1E–1H).

BJAB cells are characterized by functional BCR signaling that can be further stimulated by using a polyclonal anti-immunoglobulin antibody (aIg) (Schamel and Reth, 2000), as confirmed by aIg-induced phosphorylation of components of the BCR signal transduction pathway and aIg concentration-dependent secretion of CCL3 chemokine and interleukin-10 (IL-10) and IL-6 cytokines (data not shown). We set out to explore molecular changes induced by aIg treatment of BJAB cells at combined

(phospho)protein and transcriptome levels across a minute-to-hour timescale by applying QuRIE-seq on cells stimulated with aIg for 0, 2, 4, 6, 60, and 180 min. Data pre-processing included stringent quality control analysis and normalization, resulting in a multi-modal dataset of 4,754 cells with matched gene and (phospho)protein expression levels. Finally, the data were scaled and corrected for sequencing depth, mitochondrial content (RNA dataset), and histone H3 levels (protein dataset).

Multi-omics factor analysis

Next, we used unbiased Multi-Omics Factor Analysis (MOFA+) (Argelaguet et al., 2020) to combine the quantification of (phospho)proteins and transcripts into a single model that identifies factors explaining variation from both modalities over the full time series (Figure 1F and S2A–S2C). First, we used the computed MOFA+ factors as input for uniform manifold approximation and projection (UMAP), capturing the cellular responses to induced activation of the BCR pathway. As shown in Figure 1G, two distinct phases of cellular responses in BJAB cells are observed after short (0–2 min) and long (60–180 min) stimulation. Principal-component analysis (PCA) of the separate datasets, followed by integration of the modalities by using weighted nearest-neighbor (WNN) analysis from the Seurat v.4 package (Hao et al., 2021), confirmed these observations, showing changes at the minute timescale for the (phospho)protein dataset and the hour timescale for the transcriptome dataset in the first principal component (Figures S2D–S2H), as expected. Together, these initial observations indicate that the computed factors of QuRIE-seq measurements capture the cellular responses to a signal across modalities and timescales.

Revealing signal transduction dynamics

To explore which molecular changes underlie the BJAB cells' response to the aIg stimulus at the minute and hour timescales, we analyzed the computed MOFA+ factors in more detail and found that factors 1 and 3 most strongly correlated with the time of the treatment (Figure S2C). Factor 1 captures minute timescale changes (Figure 2A), with predominant effects on the phosphoprotein levels (Figure 2B), as confirmed by flow cytometry. Only a minor part of factor 1 is associated with the variance in the mRNA dataset, as transcriptional changes after external stimulation typically take place at the hour, not minute, timescale (Figure S2A).

Examination of the (phospho)proteins and genes with the highest loading values for factor 1 highlighted components of the BCR signaling pathway (e.g., p-CD79a, p-PLC-γ2, p-SRC, and p-SYK, *IGHM*, *IGKC*), indicating that factor 1 most likely represents the core elements of the BCR signal transduction pathway, which is activated within 2 min of BCR stimulation with aIg (Figures 2C and 2D). Surprisingly, using our unbiased approach, we identified p-JAK1 as strongly contributing to factor 1, increasing within the first 2 min after aIg addition, suggesting regulation of JAK1 phosphorylation by BCR activation (Figures 2B and 2D). Although the tyrosine kinase JAK1 has been suggested as a substrate for SYK (Patterson et al., 2015; Satpathy et al., 2015), phosphorylation of JAK1 upon BCR activation has thus far not been described. The unexpected activation of JAK1 by aIg stimulation was further confirmed with flow cytometry (Figure S3A). Notably, canonical activation of JAK1 by indirect activation of signaling through cytokines is unlikely to explain these effects, as the observed timescale of 2 min is too short for expression and/or secretion of endocrine cytokines. In addition, we found that the JAK1/2 inhibitor ruxolitinib resulted in decreased phosphorylation of the BCR pathway components SYK, Bruton's tyrosine kinase (BTK), and PLC-γ2, as well as the JAK1 substrate STAT6 (Figure S3B). This suggests a previously unidentified role for JAK1 in the BCR response to aIg stimulation revealed by QuRIE-seq. We performed additional analyses on p-JAK high versus p-JAK low cells (top and bottom 5% at each time point). At several time points, we found a common set of phosphoproteins that are differentially expressed in the high versus low cell subpopulations, including p-CD79a, p-SYK, and p-SRC, all related to BCR signaling activation. Even though no corresponding consistent differences in gene expression were found, these findings illustrate that we can distinguish cells with higher versus lower signaling responses to the stimulus within the population of cells (Figure S4).

Whereas factor 1 captures changes at the minute timescale, factor 3 distinguishes samples at the hour timescale (60 and 180 min, Figure 2E). The (phospho)proteins with the highest factor 3 loading values are p-S6, XBP1, p-p38, p-AKT, p-TOR, and p-STAT5 (Figure 2F). These (phospho)proteins have been shown to contribute to the increase in mis/unfolded proteins in activated B cells through the unfolded protein response (UPR) (Gaudette et al., 2020), activation of glycolytic energy metabolism, and cell-cycle entry. RNA features with positive factor 3 loading values (Figures 2F and 2G) are significantly enriched for gene sets associated with similar cellular functions: proteasome-mediated protein catabolic process, COPII-mediated vesicle budding, Golgi vesicle organization, and cell-cycle entry. These biological processes relate to increased protein production and energy metabolism, antibody secretion (plasma cell differentiation), and cell-cycle entry (Figure 2G). In addition to proteasome-mediated protein catabolic processes, the protein folding gene ontology (GO) term is enriched (adjusted $p = 0.021$) in the RNA dataset, consistent with the enrichment of UPR-related (phospho)proteins associated with factor 3. The expression of genes with the highest factor 3 loading, including *NEAT1*, *NPM1*, and *BTF3*, is upregulated after 60 and 180 min of BCR signaling stimulation with aIg (Figure 2H). Comparison of differentially expressed genes determined by bulk RNA sequencing and by QuRIE-seq shows similar upregulation profiles at 60 or 180 min (data not shown). In summary, the molecular changes at the hour timescale seem to be dominated by proteins and genes involved in processes related to increased protein synthesis, activated glycolysis, B cell activation, and proliferation.

Disruption of signal transduction by using ibrutinib

Finally, as QuRIE-seq allows us to map signal transduction at both the (phospho)protein and the mRNA level, we explored whether the developed platform can be used to obtain a high-resolution view of the mechanism of action of drugs. Therefore, we stimulated BJAB cells with aIg, as described above, in the presence or absence of 1 μM ibrutinib (Ibru), a small-molecule inhibitor of BTK, blocking a part of the BCR signaling (Figure S5A). We explored the effect of Ibru treatment at both minute and hour

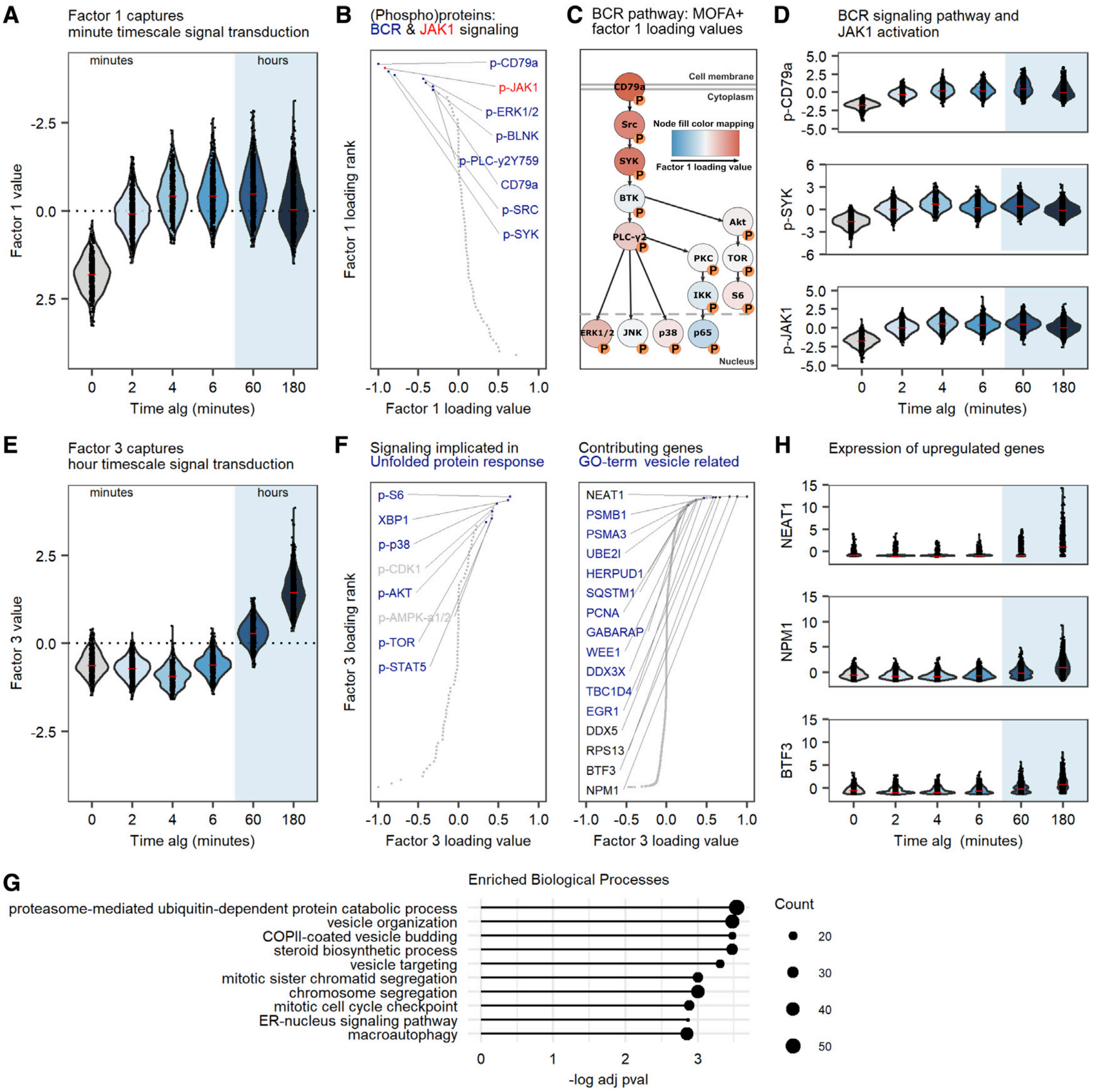

Figure 2. Multi-modal characterization of minute and hour signal transduction

(A) Violin plot of the factor 1 value as a function of the anti-immunoglobulin antibody (aIg) treatment duration. To reflect the biological intuition on increased phosphorylation upon stimulation, the y axis of factor 1 is flipped.

(B) Protein loading contributing to factor 1, ranked according to loading value. Proteins with loading value less than −0.25 are annotated in the graphs; colors highlight gene sets related to BCR signaling (blue) or JAK1 (red).

(C) BCR signaling pathway with phosphoproteins colored by MOFA+ factor 1 loading value.

(D) Scaled and normalized QuRIE-seq counts of p-CD79a, p-SYK, and p-JAK1 in BJAB cells upon stimulation with aIg.

(E) Violin plot of the factor 3 value as a function of the aIg treatment duration.

(F) Protein (left) and gene (right) loading contributing to factor 3. Proteins and genes with loading value >0.25 are indicated in the graphs. Blue color highlights proteins or genes related to the indicated biological processes.

(G) Top 10 gene ontology (GO) biological process terms enriched in factor 3 genes with positive loading values.

(H) Scaled and normalized QuRIE-seq counts of NEAT1, NPM1, and BTF3 in BJAB cells upon stimulation with aIg.

timescales, after 6 and 180 min of aIg stimulation. Using MOFA+, we computed a model explaining variation in this dataset. UMAP representation shows how aIg stimulation of BJABs in presence of Ibru affects the cellular state at both 6- and 180-min time points (Figure 3A). Factors 1 and 3 again correlate with the time of treatment with aIg and capture the short- and long-timescale changes, respectively (Figures 3B and 3C). Decreased values of factors 1 and 3 for Ibru-treated cells (Figure 3C) suggest these processes are, at least partially, BTK dependent. Indeed, phosphorylation of PLC-γ2 and BLNK is inhibited by Ibru, as were CD79a, SYK, and JAK1 (Figure S5B). The decreased phosphorylation of proteins upstream of BTK suggests the existence of a feedback loop that is affected by Ibru treatment. The expression of the top three genes with high factor

CellPress
OPEN ACCESS

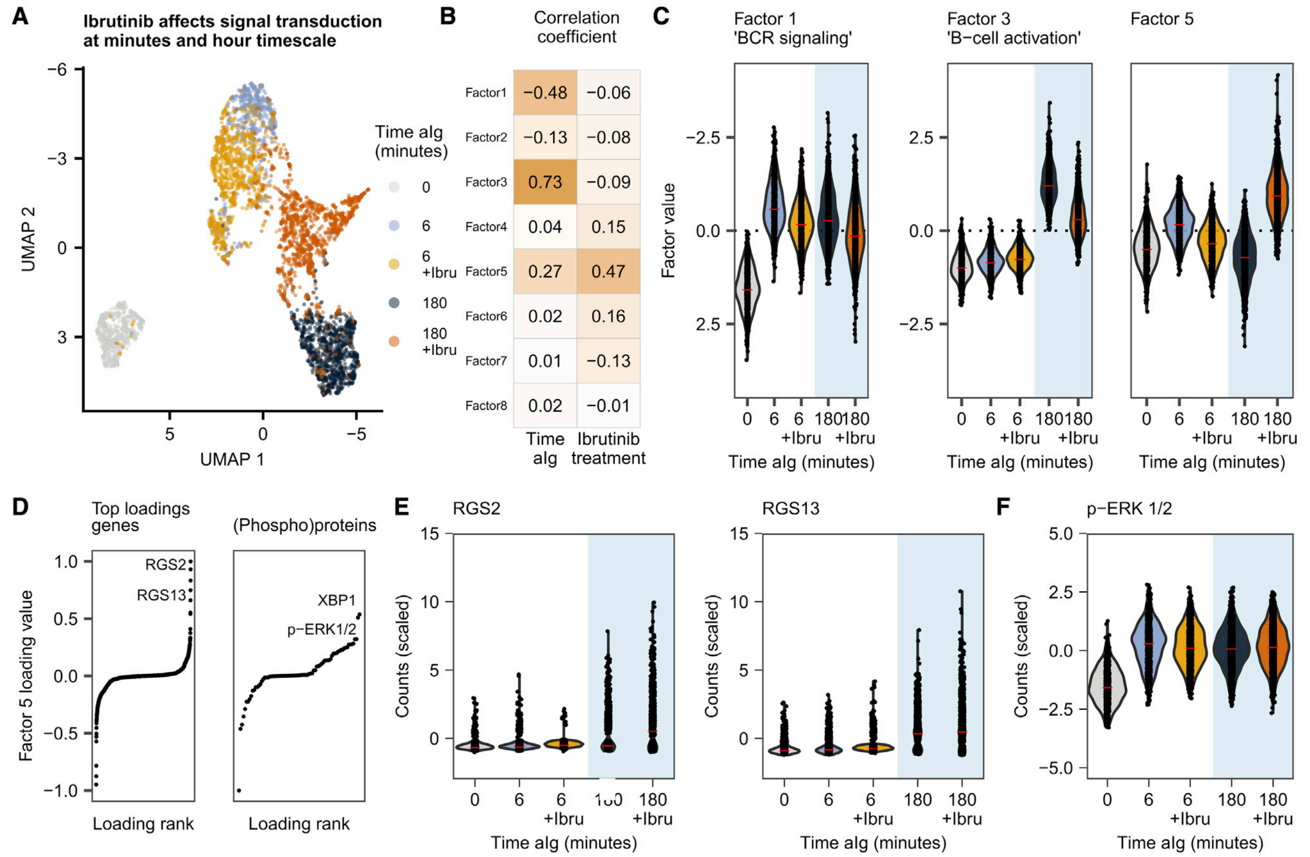

	Time aIg	Ibrutinib treatment
Factor1	−0.48	−0.06
Factor2	−0.13	−0.08
Factor3	0.73	−0.09
Factor4	0.04	0.15
Factor5	0.27	0.47
Factor6	0.02	0.16
Factor7	0.01	−0.13
Factor8	0.02	−0.01

Figure 3. Multi-modal characterization of the dynamic single-cell response to anti-immunoglobulin antibody (aIg) stimulation in presence of ibrutinib (Ibru), an inhibitor of BTK signaling

(A) UMAP embedding based on the MOFA+ factors for 4,658 BJAB cells stimulated with aIg for different durations: 0, 6, and 180 min with or without Ibru inhibition.
(B) Pearson correlation coefficient by each factor correlating with aIg stimulation duration and with Ibru inhibition.
(C) Violin plots of factor 1, 3, and 5 values as a function of the aIg treatment duration with or without Ibru inhibition.
(D) Gene and protein loading contributing to factor 5, ranked according to the loading value.
(E) Violin plots of *RGS2* and *RGS13* gene normalized and scaled QuRIE-seq counts.
(F) Violin plot of p-ERK 1/2 normalized and scaled QuRIE-seq counts.

3 loading shows diminished upregulation after 180 min of treatment with aIg (Figure S5C). To identify additional effects of BTK inhibition, we note that factor 5 most strongly correlates (Pearson correlation = 0.47) with Ibru treatment and shows increased values after 180 min of stimulation with aIg for Ibru-inhibited cells compared with cells without Ibru inhibition (Figures 3B and 3C).

GO-term analysis shows that features highly contributing to this factor in the positive loadings are two negative regulators of G-protein-coupled signaling, *RGS2* and *RGS13*, for the RNA dataset (GO: 0045744, adjusted p = 0.22). RGS2 and RGS13 expression after disturbed BCR activation might relate to the germinal center (GC) phenotype of BJAB cells, where signaling through the G-protein-coupled receptors CXCR4 and CXCR5 orchestrates GC dynamics (Wu et al., 2019). Furthermore, in the protein dataset, p-ERK 1/2 contributes, albeit modestly, to factor 5 (Figures 3D–3F). Surprisingly, ERK 1/2 phosphorylation is maintained independent of Ibru-mediated BTK inhibition (Figure 3F), which supports the notion that Ibru only partially blocks B cell signal transduction. In line with this, Ibru does indeed inhibit, in a dose-dependent manner, secretion of IL-10 and CCL3, but not IL-6 (Figure S5D), supporting the notion of BTK-dependent and -independent aIg-induced activation of BJAB cells. These precursory findings illustrate the potential of QuRIE-seq to study the complexity of inhibitory drug effects on signal transduction.

DISCUSSION

In conclusion, QuRIE-seq combined with a time series of single-cell intracellular epitope and transcriptome data provide a powerful method to study multi-modal intracellular signal transduction at multiple timescales and characterize the mechanism of action of drugs on signal transduction. Here, we used an antibody panel targeting 80 (phospho)proteins, but we believe there are no biophysical or biochemical limitations to expanding this panel to include 500+ target epitopes. Targeting a large number of (phospho)proteins can reveal previously unidentified signaling

networks, such as the involvement of JAK1 in BCR signaling or the BTK-independent activation of ERK 1/2. Although we have exploited droplet-based microfluidics to implement QuRIE-seq, we envision future adaptation to high-throughput plate-based methods such as sci-Plex (Zheng et al., 2017) and Seq-Well (Gierahn et al., 2017). The MOFA+ framework allowed us to analyze both modalities simultaneously, with clear and interpretable results. With an increasing number of multi-modal single-cell technologies becoming available, complementary analysis methods are rapidly being developed, and other options will be available for analyzing our QuRIE-seq data. For example, we tested WNN (implemented in Seurat v.4; Hao et al., 2021) analysis of our data, and combined PCA and WNN analysis shows results very similar to those of MOFA+ in terms of resolving the time-dependent manner of phosphoprotein activation at short timescales, as well as transcriptional changes at later time points. The combination of customized antibody panels, the ability to detect changes with a high temporal resolution, and the multi-omic readout of the effect of inhibitory drugs leads us to anticipate a broad implementation of QuRIE-seq in fundamental signaling studies and drug development research.

Limitations of the study

QuRIE-seq relies on binding of DNA-tagged antibodies to membrane and intracellular epitopes. Cell cross-linking might change protein conformation and thus affect the binding of antibodies, limiting our ability to quantify protein levels of those targets. In this study, we used some 80 antibodies in our panel. Expansion of the panel brings significant additional costs.

Our initial studies on primary B cells indicated that mRNA extraction was insufficient to obtain a good-quality mRNA library. Further improvements in the reversible cross-linking chemistry are required.

STAR★METHODS

Detailed methods are provided in the online version of this paper and include the following:

- KEY RESOURCES TABLE
- RESOURCE AVAILABILITY
  - Lead contact
  - Materials availability
  - Data and code availability
- EXPERIMENTAL MODEL
- METHOD DETAILS
  - Antibody labeling
  - BJAB cells preparation
  - Primary B-cells preparation
  - Flow cytometry analysis
  - ELISA
  - Bulk mRNA sequencing
  - Microfluidic device fabrication for single-cell barcoding
  - Microfluidic single-cell barcoding
  - Size separation of mRNA and protein libraries
  - mRNA library preparation
  - Protein libraries preparation
  - Bulk mRNA Sequencing data analysis
  - From FASTQ files to count tables
  - Quality control, filtering and normalization
- QUANTIFICATION AND STATISTICAL ANALYSIS
  - Multi-omics Factor Analysis + (MOFA+) Model on time series of alg stimulation
  - Multi-omics Factor Analysis + (MOFA+) model on time-points including Ibrutinib data

SUPPLEMENTAL INFORMATION

Supplemental information can be found online at https://doi.org/10.1016/j.crmeth.2021.100070.

ACKNOWLEDGMENTS

We thank Sun Tian for assistance with the analysis of RNA bulk sequencing. We acknowledge financial support from Aduro Biotech Europe and Radboud University, Nijmegen.

AUTHOR CONTRIBUTIONS

W.T.S.H., K.W.M., and H.v.E. supervised the research. F.R., E.v.B., and K.M. designed and performed the QuRIE-seq experiment. E.v.B. performed the flow cytometry, ELISA, and bulk sequencing experiments. F.R. and J.A.G.L.v.B. analyzed the sequencing results. All authors actively contributed to writing and improving the manuscript and approved the final version.

DECLARATION OF INTERESTS

The authors declare no competing interests.

Received: January 20, 2021
Revised: May 6, 2021
Accepted: August 6, 2021
Published: September 15, 2021

REFERENCES

Argelaguet, R., Arnol, D., Bredikhin, D., Deloro, Y., Velten, B., Marioni, J.C., and Stegle, O. (2020). MOFA+: A statistical framework for comprehensive integration of multi-modal single-cell data. Genome Biol. *21*, 111.

Blischak, J.D., Carbonetto, P., and Stephens, M. (2019). Creating and sharing reproducible research code the workflow way [version 1; peer review: 3 approved]. F1000Res. *8*, 1749.

Gaudette, B.T., Jones, D.D., Bortnick, A., Argon, Y., and Allman, D. (2020). mTORC1 coordinates an immediate unfolded protein response-related transcriptome in activated B cells preceding antibody secretion. Nat. Commun. *11*, 723.

Gerlach, J.P., van Buggenum, J.A.G., Tanis, S.E.J., Hogeweg, M., Heuts, B.M.H., Muraro, M.J., Elze, L., Rivello, F., Rakszewska, A., van Oudenaarden, A., et al. (2019). Combined quantification of intracellular (phospho-)proteins and transcriptomics from fixed single cells. Sci. Rep. *9*, 1–10.

Gierahn, T.M., Wadsworth, M.H., Hughes, T.K., Bryson, B.D., Butler, A., Satija, R., Fortune, S., Christopher Love, J., and Shalek, A.K. (2017). Seq-Well: portable, low-cost RNA sequencing of single cells at high throughput. Nat. Methods *14*, 395–398.

Hafemeister, C., and Satija, R. (2019). Normalization and variance stabilization of single-cell RNA-seq data using regularized negative binomial regression. Genome Biol. *20*, 296.

Hao, Y., Hao, S., Andersen-Nissen, E., Mauck, W.M., Zheng, S., Butler, A., Lee, M.J., Wilk, A.J., Darby, C., Zager, M., et al. (2021). Integrated analysis of multimodal single-cell data. Cell *184*, 3573–3587.e29.

Klein, A.M., Mazutis, L., Akartuna, I., Tallapragada, N., Veres, A., Li, V., Peshkin, L., Weitz, D.A., and Kirschner, M.W. (2015). Droplet barcoding

CellPress
OPEN ACCESS

for single-cell transcriptomics applied to embryonic stem cells. Cell *161*, 1187–1201.

Mimitou, E.P., Cheng, A., Montalbano, A., Hao, S., Stoeckius, M., Legut, M., Roush, T., Herrera, A., Papalexi, E., Ouyang, Z., et al. (2019). Multiplexed detection of proteins, transcriptomes, clonotypes and CRISPR perturbations in single cells. Nat. Methods *16*, 409–412.

Patterson, H.C., Gerbeth, C., Thiru, P., Vögtle, N.F., Knoll, M., Shahsafaei, A., Samocha, K.E., Huang, C.X., Harden, M.M., Song, R., et al. (2015). A respiratory chain controlled signal transduction cascade in the mitochondrial intermembrane space mediates hydrogen peroxide signaling. Proc. Natl. Acad. Sci. U S A *112*, E5679–E5688.

Peterson, V.M., Zhang, K.X., Kumar, N., Wong, J., Li, L., Wilson, D.C., Moore, R., Mcclanahan, T.K., Sadekova, S., and Klappenbach, J.A. (2017). Multiplexed quantification of proteins and transcripts in single cells. Nat. Biotechnol. *35*, 936–939.

Satpathy, S., Wagner, S.A., Beli, P., Gupta, R., Kristiansen, T.A., Malinova, D., Francavilla, C., Tolar, P., Bishop, G.A., Hostager, B.S., et al. (2015). Systems-wide analysis of BCR signalosomes and downstream phosphorylation and ubiquitylation. Mol. Syst. Biol. *11*, 810.

Schamel, W.W.A., and Reth, M. (2000). Monomeric and oligomeric complexes of the B cell antigen receptor. Immunity *13*, 5–14.

Shannon, P., Markiel, A., Ozier, O., Baliga, N.S., Wang, J.T., Ramage, D., Amin, N., Schwikowski, B., and Ideker, T. (2003). Cytoscape: a software Environment for integrated models of biomolecular interaction networks. Genome Res. *13*, 2498–2504.

Sinha, N., Subedi, N., Wimmers, F., Soennichsen, M., and Tel, J. (2019). A pipette-tip based method for seeding cells to droplet microfluidic platforms. J. Vis. Exp. https://doi.org/10.3791/57848.

Stoeckius, M., Hafemeister, C., Stephenson, W., Houck-Loomis, B., Chattopadhyay, P.K., Swerdlow, H., Satija, R., and Smibert, P. (2017). Simultaneous epitope and transcriptome measurement in single cells. Nat. Methods *14*, 865–868.

Stuart, T., Butler, A., Hoffman, P., Hafemeister, C., Papalexi, E., Mauck, W.M., Hao, Y., Stoeckius, M., Smibert, P., and Satija, R. (2019). Comprehensive integration of single-cell data. Cell *177*, 1888–1902.e21.

Wu, B.X., Zhao, L., and Zhang, X. (2019). CXCR4 and CXCR5 orchestrate dynamic germinal center reactions and may contribute to the pathogenesis of systemic lupus erythematosus. Cell. Mol. Immunol. *16*, 724–726.

Yu, G., Wang, L.G., Han, Y., and He, Q.Y. (2012). ClusterProfiler: an R package for comparing biological themes among gene clusters. Omi. A. J. Integr. Biol. *16*, 284–287.

Zheng, G.X.Y., Terry, J.M., Belgrader, P., Ryvkin, P., Bent, Z.W., Wilson, R., Ziraldo, S.B., Wheeler, T.D., McDermott, G.P., Zhu, J., et al. (2017). Massively parallel digital transcriptional profiling of single cells. Nat. Commun. *8*, 14049.

Zhu, C., Preissl, S., and Ren, B. (2020). Single-cell multimodal omics: the power of many. Nat. Methods *17*, 11–14.

Zilionis, R., Nainys, J., Veres, A., Savova, V., Zemmour, D., Klein, A.M., and Mazutis, L. (2017). Single-cell barcoding and sequencing using droplet microfluidics. Nat. Protoc. *12*, 44–73.

STAR★METHODS

KEY RESOURCES TABLE

REAGENT or RESOURCE	SOURCE	IDENTIFIER
Antibodies		
Antibodies used for immunostaining of cells prior to microfluidic encapsulation are listed in Table S1	See Table S1	N/A
Antibodies used for flow cytometry analysis of BJABs are listed in Table S2	See Table S2	N/A
Biological samples		
Buffy coat	Sanquin, The Netherlands	N/A
Chemicals, peptides, and recombinant proteins		
RPMI medium	Thermo Fisher Scientific, USA	21875034
Ibrutinib (PCI-32765)	Selleck Chemicals, USA	S2680
$F(ab')_2$ Fragment Goat Anti-Human IgA + IgG + IgM (H+L)	Jackson ImmunoResearch, USA	109-006-064
dibenzocyclooctyne-S-S-N-hydroxysuccinimidyl ester	Sigma Aldrich, USA	761532
dithiobis(succinimidyl propionate) (DSP)	Thermo Fisher Scientific, USA	22585
succinimidyl 3-(2-pyridyldithio)propionate (SPDP)	Thermo Fisher Scientific, USA	10220264
Barcoded hydrogel beads	1CellBio, USA	N/A
Superscript III	Thermo Fisher Scientific, USA	12574026
RAN surfactant	RAN Biotechnologies, USA	008-FluoroSurfactant-5wtH-20G
PrimeScript RT	Takara, USA	RR037
Critical commercial assays		
HiScribe™ T7 High Yield RNA Synthesis Kit	New England Biolabs, USA	E2040S
Deposited data		
Raw and analyzed data	This paper	GEO: GSE162461
Experimental models: Cell lines		
BJAB cell line	DSMZ, Germany	ACC 757
Oligonucleotides		
Primers used for library preparation are listed in Table S3	Biolegio, The Netherlands	N/A
Software and algorithms		
inDrop script to generate count tables from the FASTQ files	Klein et al., 2015	https://github.com/indrops/indrops
WorflowR	Blischak et al., 2019	https://github.com/jdblischak/workflowr
MOFA+	Argelaguet et al., 2020	https://biofam.github.io/MOFA2/index.html
FlowJo	BD Bioscience, USA	N/A
Rstudio	*RStudio: Integrated Development for R. RStudio, PBC, Boston, MA*	https://www.rstudio.com/
Seurat	Hao et al., 2021	https://satijalab.org/seurat/
Analysis code to generate figures in the manuscript	This paper	https://zenodo.org/record/5082775 https://doi.org/10.5281/zenodo.5082775
EnrichGO from clusterprofiler	Yu et al., 2012	http://yulab-smu.top/clusterProfiler-book/

CellPress
OPEN ACCESS

RESOURCE AVAILABILITY

Lead contact

Further information and requests for resources and reagents should be directed to and will be fulfilled by the lead contact, Prof. Dr. Wilhelm T. S. Huck (w.huck@science.ru.nl).

Materials availability

This study did not generate new unique reagents.

Data and code availability

- Single-cell RNA-seq data have been deposited at GEO and are publicly available as of the date of publication. Accession numbers are listed in the key resources table. Flow cytometry data reported in this paper will be shared by the lead contact upon request.
- All original code has been deposited at Zenodo and is publicly available as of the date of publication. DOIs are listed in the key resources table.
- Any additional information required to reanalyze the data reported in this paper is available from the lead contact upon request.

EXPERIMENTAL MODEL

The BJAB cell line used in this study were obtained from DSMZ, Germany. The cell line was established in 1973 from the inguinal tumor of an EBV-negative 5-year-old African girl with Burkitt lymphoma at the preterminal stage; although the tumor tissue was experimentally exposed to EBV, neither EBV sequences nor EBV antigens were detectable in the established cell line. Exome and RNA sequence data are available DSMZ (https://www.dsmz.de/collection/catalogue/details/culture/ACC-757).

METHOD DETAILS

Antibody labeling

All antibodies (Abs) were purchased purified (see Table S1 for a full list of the Abs used). Abs used in the QuRIE-seq experiment were first validated using flow cytometry.

QuRIE-seq requires the use of DNA-tagged Abs for staining cells prior to encapsulation to characterize the single-cell proteome. In the following is briefly detailed the DNA conjugation strategy to the Abs which made us of standard NHS chemistry. First, Abs were buffer exchanged into 0.2M $NaHCO_3$ (pH 8.3) (Sigma Aldrich, USA) using Zeba^{TM} Spin Desalting Columns, 40K MWCO (Thermo Fisher Scientific, USA). Then, dibenzocyclooctyne-S-S-N-hydroxysuccinimidyl ester (Sigma Aldrich, USA) was resuspended in DMSO (Sigma Aldrich, USA), added in a 10x molar excess to the Abs and incubated for 2 hours at room temperature. Abs were then buffer exchanged into PBS (Thermo Fisher Scientific, USA) and excess linker molecules were removed using 30K Amicon centrifuge units (Merck, USA). Subsequently, 5' Azide modified oligos (Biolegio, The Netherlands) were added in a 3x molar excess to the chemically modified Abs and incubated for sixteen hours at 4°C in dark. After incubation, Abs were buffer exchanged into PBS (Thermo Fisher Scientific, USA) containing 0.05% sodium azide (Sigma Aldrich, USA) and 0.1mM EDTA (Sigma Aldrich, USA), and excess oligos were removed using 100K Amicon centrifuge filters (Merck, USA). After labelling, DNA-tagged Abs were stored at 4°C. Labelling efficiency was determined by non-reducing SDS page gel analysis where 0.5 μg antibody was diluted in 1x Laemmli sample buffer (Bio-Rad, USA) and loaded on a mini-PROTEAN TGX stain-free gel (Bio-Rad, USA). Gels were imaged on a ChemiDoc Touch imaging system (Bio-Rad, USA).

BJAB cells preparation

BJAB cells were obtained from DSMZ, Germany (ACC 757). Cells were cultured in RPMI medium (Thermo Fisher Scientific, USA) supplemented with 20% FBS (Biowest, France) and 1% Penicillin-Streptomycin (Thermo Fisher Scientific, USA). Before stimulation, cells were harvested, centrifuged at 200 g for 5 minutes and re-suspended in complete medium at a concentration of $3*10^6$ cells/ml. For each time point, 500 μl cell suspension was dispensed in 3 ml FACS tubes (BD biosciences, USA). For cells treated with inhibitor 1 μM Ibrutinib (Selleck Chemicals, USA) was added directly to the cell suspension. Subsequently, cells were allowed to rest in the complete medium for one hour at 37°C with or without an inhibitor. Cells were then stimulated with 10 μg/ml $F(ab')_2$ Fragment Goat Anti-Human IgA + IgG + IgM (H+L) (Jackson ImmunoResearch, USA) for the indicated time points. After incubation, cells were immediately fixated by adding two times concentrated fixative (5 mM dithiobis(succinimidyl propionate) (DSP) (Thermo Fisher Scientific, USA) and 5 mM succinimidyl 3-(2-pyridyldithio)propionate (SPDP) (Thermo Fisher Scientific, USA) in PBS (Thermo Fisher Scientific, USA), and incubated for one hour at room temperature. Fixated cells were quenched and permeabilized with 100 mM Tris-HCl pH 7.5, 150 mM NaCl and 0.1% Triton X100 (Thermo Fisher Scientific, USA) for 10 minutes at room temperature. After permeabilization, cells were washed once and blocked for 45 minutes in

0.5X protein-free blocking buffer (Thermo Fisher Scientific, USA) with 0.2 mg/ml dextran sulfate (Sigma Aldrich, USA) and 0.5 U/ml RNasin plus (Promega, USA) in PBS (Thermo Fisher Scientific, USA). Cells were stained in a blocking buffer containing DNA-tagged antibodies (Ab list used for QuRIE-seq encapsulation in Table S1, Abs used for flow cytometry in Table S2) for one hour at room temperature. Following staining, cells were washed twice with the blocking buffer and re-suspended in PBS containing 5 mg/ml BSA (Thermo Fisher Scientific, USA) and 0.5 U/ml RNsing plus (Promega, USA). Cells were sorted using the BD Biosciences FACS Melody, based on singlets, into protein LoBind tubes (Eppendorf, Germany). Just before encapsulation, each cell suspension was brought to 10^5 cells/ml and supplemented with 15% (v/v) Optiprep (Sigma Aldrich, USA).

Primary B-cells preparation

Buffy coats (Sanquin, The Netherlands) were firstly divided into two 50 ml tubes, then 750μl RosetteSep B-cell enrichment cocktail (Stemcell, Canada) was added, and blood was incubated for 20 min on a roller bench at room temperature. Subsequently, B-cell enriched PBMCs were isolated by Ficoll density gradient separation (GE Healthcare, USA). Enriched PBMCs were washed twice in PBS (Thermo Fisher Scientific, USA) containing 2mM EDTA (Lonza, Switzerland). Total B-cells were further isolated by the B-cell isolation kit II (MACS, Miltenyi Biotec, Germany) according to manufactures instructions. Purified B-cells were resuspended at a concentration of $4*10^6$ cells/ml in RPMI (Thermo Fisher Scientific, USA) supplemented with 1% Penicillin-Streptomycin (Thermo Fisher Scientific, USA) and 50 μM 2-Mercaptoethanol (Thermo Fisher Scientific, USA). 200μl of cell suspension was pipetted into FACS tubes (BD Bioscience, USA) and cells were allowed to rest for one hour at 37°C. Subsequently, cells were stimulated with 10 μg/ml $F(ab')_2$ Fragment Goat Anti-Human IgA + IgG + IgM (H+L) (Jackson ImmunoResearch, USA) for the indicated time points. After incubation, cells were immediately fixed in an equal volume 2x concentrated fixative, permeabilized and labelled as described for the BJAB cells above.

Flow cytometry analysis

DSP- and SPDP-fixed and permeabilized BJAB cells were centrifuged at 800 g and resuspended in PBS (Thermo Fisher Scientific, USA) supplemented with 0.1% BSA (Thermo Fisher Scientific, USA) containing the indicated Abs, and incubated for one hour at room temperature in dark (Table S2 shows different Abs used for flow cytometry analysis). After incubation, cells were washed three times with PBS containing 0.1% BSA and resuspended in PBS with 0.1% BSA. Finally, cells were measured on a FACSverse (BD Bioscience, USA). Flow cytometry results were analysed using FlowJo software (BD Bioscience, USA) (10000 cells we measured per sample).

ELISA

BJAB cells were cultured in round bottom 96-wells plates at a concentration of 10^5 cells/well in a volume of 200 μl. Cells were stimulated with 0-20 μg/ml $F(ab')_2$ Fragment Goat Anti-Human IgA + IgG + IgM (H+L) (Jackson ImmunoResearch, USA) and incubated for 16 hours at 37°C. The supernatant was collected and analysed for the presence of IL6, IL10 and CCL3 using uncoated ELISA kit (Thermo Fisher Scientific, USA). ELISA was performed according to the manufacturer's instructions.

Bulk mRNA sequencing

BJAB cells were harvested, centrifuged and resuspended in complete medium at $0.5*10^6$ cells/ml. Thereafter, 4 ml of cells were seeded in T25 flasks (Thermo Fisher Scientific, USA), and cells were incubated for 1 hour at 37 °C. After incubation, the cells were stimulated with 10 μg/ml $F(ab')_2$ Fragment Goat Anti-Human IgA + IgG + IgM (H+L) (Jackson ImmunoResearch, USA) for the indicated time points. After stimulation, cells were immediately collected on ice and centrifuged at 200 g for 5 minutes at 4°C. Supernatant was collected and stored at -20°C for further analysis and the cell pellet was resuspended in the lysis buffer RLT (Qiagen, The Netherlands). Cell lysates were stored at -80°C until further use. Library preparation and sequencing was performed by Macrogen (Macrogen, Korea).

Microfluidic device fabrication for single-cell barcoding

Microfluidic devices were fabricated by using photo- and soft lithography based on the AutoCAD files with channel designs that were kindly provided by Dr. Linas Mazutis (Klein et al., 2015). Silicon wafers were spin-coated with a uniform layer of SU8-2050 photoresist (MicroChem Co., USA), soft-baked, UV exposed through transparency mask (JD Phototools, UK), baked again post-exposure, developed and hard-baked according to manufacturer's protocol (MicroChem Co., USA). After production, the height of the structures was measured by Dektak profilometer (the average height ~70 μm). The obtained wafers were used as masters for the PDMS devices. PDMS prepolymer and crosslinking agent were mixed at 10:1 ratio (w/w) and poured on the master bearing the microchannel structure, degassed for 30 minutes and cured at 65 °C for at least 2 hours. Subsequently, the PDMS replica was cut out from the master and the holes were punched at inlet and outlet ports using a biopsy puncher of 1 mm inner diameter (pfm medical, USA). Thereafter, glass slides were carefully washed with a mixture of soap and water, and then both the replicas and the glass slides were rinsed with ethanol. The clean replicas were bonded to the glass slides after oxygen plasma treatment (Femto, Diener electronic, Germany). The channels of the devices were rendered hydrophobic by silanization with 2% 1H,1H,2H,2H-perfluoro-octlytriethoxysilane (Sigma-Aldrich, USA) in FC-40 (Sigma Aldrich, USA). The devices were incubated at 95 °C overnight and afterward stored at room temperature until further usage for droplet production.

Microfluidic single-cell barcoding

The chip design described by Klein et al.[6] for single-cell barcoding was used, with four inlets (reverse transcription mix, beads, cell suspension and oil phase with surfactant). The microfluidic single-cell barcoding experimental workflow was followed as described by (Zilionis et al., 2017) with some modifications. Prior to microfluidic encapsulation, the dispersed and continuous phases used for emulsification were prepared. Barcoded hydrogel beads (1CellBio, USA) were spun down, and mixed with 2X concentration bead mix (0.2% vol/vol Triton X-100 and 2x First Strand buffer) and centrifuged at 5000 g for 2 minutes. The supernatant was removed and the concentrated beads were loaded into the tubing with a previously made air spacer to avoid spreading of beads in HFE-7500 fluorinated fluid (3M) loaded syringe. The cell suspension mixed with 15% (v/v) Optiprep (Sigma-Aldrich, USA) was loaded using a pipette-tip based method for seeding cells to droplet microfluidic platforms, described elsewhere (Sinha et al., 2019). The reverse transcription (RT) mix comprising 1.3X RT premix $MgCl_2$ (1M), DTT (1M), RNAse OUT (40U/μL), Superscript III (200U/μL) and RNAse free water (Ambion, Thermo Fisher Scientific, USA) was loaded into the tubing. The tubing with the RT mix was wrapped around the ice pack to prevent the increase of temperature and deactivation of enzymes. The oil phase consisted of 3x (v/v) diluted 10% (w/w) RAN (RAN Biotechnologies, USA) in HFE-7500 oil. Solutions were introduced into the microfluidic chip using neMESYS (Cetoni GmbH, Germany) syringe pumps. First, the beads were injected at ~200 μL/h. As soon as the beads appeared at the inlet port, the flow was decreased down to ~20 μL/h, and cell suspension, RT mix, oil spacer were loaded at flow rates ~150 μL/h. When all reagent started to flow through the channels, the final flow rates of the beads, cell suspension, RT mix, oil phase were established ~15-20 μL/h (the flow was tuned during the experiment), 100 μL/h, 100 μL/h, and 90 μL/h, respectively, to produce 2 nl drops. Closely packed barcoding beads were co-encapsulated with single cells in nanolitre droplets, reaching 60-90% cell barcoding efficiency.

After microfluidic emulsification, the cells were de-crosslinked by the action of DTT present in the RT mix which reduced the disulphide bonds present and the barcoding primers were released from the hydrogel beads by 8-minute UV exposure at 10 mW/cm2 from a photolithography machine (ABN Inc, USA). Subsequently, the poly(dT) tail of the barcoding primers annealed both to the poly(A) tail of the mRNA and the poly(dA) tail of the Ab tags and the single cells-derived material was barcoded by reverse transcription: incubation of 2 hours at 50°C, followed by 15 minutes at 70 °C. The emulsion was then broken by adding 3 μL of 20% (v/v) PFO on the top of the emulsion, and then 40 μL of HFE-7500 fluorinated fluid (3M). The samples were stored until further use at -80°C.

Size separation of mRNA and protein libraries

After microfluidic single-cell barcoding, samples were thawed, centrifuged at 4°C for 5 minutes at 19000 g to pellet cell debris, then the aqueous post-RT material (top layer) was separated from the bottom oil layer and transferred to a nucleic acid purification column (Corning Costar Spin-X column, 0.45 μm, Sigma-Aldrich, USA). Tubes were centrifuged for 1 minute at 16000 g at 4°C. Subsequently, 100 μL of digestion mix was added per each 70 μL of the sample (final concentrations were 0.5x FastDigest Buffer (Thermo Fisher Scientific, USA) and 0.6 U/μL ExoI (Thermo Fisher Scientific, USA)) and incubated for 30 minutes at 37°C. After digestion of unused primers and primer dimers, the mix was size separated using 0.6x volume of AMPure XP magnetic beads (Beckman Coulter, USA). The short fragments (the proteomic library that was retained in the supernatant) were separated from the long fragments (the transcriptomic library kept by beads) and each library was then processed independently.

mRNA library preparation

After the size separation of the mRNA library from the protein library, the mRNA library was processed for second-strand synthesis (SSS) and linear amplification by In Vitro Transcription (IVT). Briefly, 2 μL of 10x SSS buffer and 1 μL of SSS Enzyme (NEBNext mRNA Second Strand Synthesis Module, New England Biolabs, USA) were added to the 17 μL of the purified sample, mixed, and incubated at 16 °C for 1.5 hour, and 20 min at 65 °C. Subsequently, 60 μL of IVT mix was added to each of the 20 μL SSS product, and incubated at 37 °C for 15 hours. The IVT reaction was composed by 1x reaction buffer, 10mM each of ATP, CTP, GTP and UTP, and 10x diluted T7 RNA Polymerase mix (HiScribe™ T7 High Yield RNA Synthesis Kit, New England Biolabs, USA). After linear amplification, the amplified RNA material was purified with 1.3x AMPure XP magnetic beads (Beckman Coulter, USA), and fragmented by adding 1 μL of 10X RNA fragmentation Reagents (Thermo Fisher Scientific, USA) to 10ul of purified IVT, mixing and immediately incubating at 70 °C for exactly 3 minutes. Thereafter, the mix was transferred on ice and 34 μl of STOP mix (8.9 μl nuclease-free water, 24 μl AMPure XP magnetic beads, and 1.1 μl STOP solution) were added to it. Purification using AMPure XP magnetic bead was followed by sample elution in 8 μl nuclease-free water. The following step of the library preparation consisted of the reverse transcription (RT) of the amplified RNA material using random hexamers. This was performed by first addition of 1 μL of 10 nM of each dNTP (Thermo Fisher Scientific, USA) and 2 μL of 100 μM of PE2-N6 primer (Biolegio, The Netherlands), and incubation of the mix at 70 °C for 3 minutes, followed by cooling down the mixture on ice. Next, 3.5 μL nuclease-free water was added to 4 μL of 5x PrimeScript buffer (Takara, USA), 1 μL RNAse OUT (Thermo Fisher Scientific, USA) and 0.5 μL PrimeScript RT (Takara, USA), and the reaction mix was incubated at 30 °C for 10 minutes, followed by 42 °C for 1 hour and by 15 minutes at 70 °C. The RT product was then purified with 1.2x AMPure XP magnetic beads and eluted in 10 μL of nuclease-free water and amplified by PCR. The number of required cycles was determined by qPCR using 0.5 μL of purified material with the addition of 6.5 μL nuclease-free water, 10 μL of 2x Kapa HiFi HotStart PCR mix (Roche, Switzerland), 1 μL of 20x EvaGreen Dye (Thermo Fisher Scientific, USA), and 2 μL of PE1/PE2 primer mix (5 μM each) (Biolegio, The Netherlands). Final material was obtained by mixing 0.5 μL of nuclease-free water, 12.5 μL of 2x Kapa HiFi HotStart PCR mix (Roche, Switzerland), and 2 μL of PE1/PE2 primer mix (5 μM each) (Biolegio, The Netherlands) and the 9.5 μL of the sample. The PCR thermal cycling was as follows: 2 minutes at 98 °C, followed by 2 cycles of: 20 seconds at 98 °C, 30 seconds at

55 °C and 40 seconds at 72 °C, followed by x cycles of (x = number previously determined by qPCR): 20 seconds at 98 °C, 30 seconds at 65 °C and 40 seconds at 72 °C, and terminated with 5 minute-incubation at 72 °C. After PCR, the sample was purified by the addition of 0.7x AMPure XP magnetic beads and eluted in 10 μl of water. The quality of the final libraries was verified by ds-DNA concentration measurement using Qubit ds-DNA High Sensitivity assay (Thermo Fisher Scientific, USA) and BioAnalyzer (Agilent 2100, USA), following the manufacturer's instructions. If the quality of the samples was sufficient, the mRNA libraries were sequenced together with the protein libraries on with NextSeq500 (targeting 50 million reads per sample). The sequences of the primers used for the library preparations and sequencing are shown in Table S3.

Protein libraries preparation

After size separation of the protein and mRNA libraries, the protein library was amplified by IVT reaction. 20 μL of the product was mixed with 60 μL of IVT mix and amplified for 15 hours at 50 °C. The IVT mix was composed of 1x reaction buffer, 10 mM each dNTP (ATP, CTP, GTP and UTP), and 10x diluted T7 RNA Polymerase mix (HiScribe^{TM} T7 High Yield RNA Synthesis Kit, New England Biolabs, USA). After linear amplification, the sample was purified by the addition of 1.5x AMPure XP magnetic beads and eluted in 20 μl of water. 10 μl of the sample were stored at -80 °C as post-IVT backup and the other 10 μl were mixed with 1 μL of 10 nM of each dNTP (Thermo Fisher Scientific, USA), 1 μL of 10 μM PE2-NNNN-Next1 primer (Biolegio, The Netherlands), and 1 μL of 10 μM PE2-NNNN-BioHash2 primer (Biolegio, The Netherlands) and reversely transcribed by incubation for 3 minutes at 70 °C followed by cooling down the mixture on ice. Next, 1.5 μL nuclease-free water, 4 μL of 5x PrimeScript buffer (Takara, USA), 1 μL RNAse OUT (Thermo Fisher Scientific, USA) and 0.5 μL of 200 U/μL PrimeScript RT (Takara, USA) was added to the reaction mix that was incubated: at 30 °C for 10 minutes, followed by 42 °C for 1 hour and by 15 minutes at 70 °C. After reverse transcription, the product was purified with 1.5x AMPure XP magnetic beads and eluted in 10 μL of nuclease-free water. Subsequently, the sample was PCR amplified after prior determination of the number of cycles needed by qPCR: 0.5 μL of purified material was mixed with 6.5 μL nuclease-free water, 10 μL of 2x Kapa HiFi HotStart PCR mix (Roche, Switzerland), 1 μL of 20X EvaGreen Dye (Thermo Fisher Scientific, USA), and 2 μL of PE1/PE2 primer mix (5 μM each) (Biolegio, The Netherlands). Then the PCR reaction was started by adding: 0.5 μL of nuclease-free water, 12.5 μL of 2X Kapa HiFi HotStart PCR mix (Roche, Switzerland), and 2.5 μL of PE1/PE2 primer mix (5 μM each) (Biolegio, The Netherlands) to the 9.5 μL of the sample. The PCR thermal cycling was as follows: 2 minutes at 98 °C, followed by 2 cycles of: 20 seconds at 98 °C, 30 seconds at 55 °C and 40 seconds at 72 °C, followed by x cycles of (x = number previously determined by qPCR): 20 seconds at 98 °C, 30 seconds at 65 °C and 40 seconds at 72 °C, and terminated with 5 minute-incubation at 72 °C. PCR amplified product was purified by addition of 1.2X AMPure XP magnetic beads, eluted in 10 μl water and its quality was verified by ds-DNA concentration measurement with Qubit ds-DNA High Sensitivity assay (Thermo Fisher Scientific, USA) and BioAnalyzer (Agilent 2100, USA) characterization. If the quality of the samples was high enough, the protein libraries were sequenced together with the mRNA libraries on with NextSeq500 (targeting 50 million reads per sample). The sequences of the primers used for the library preparations and sequencing are shown in Table S3.

Bulk mRNA Sequencing data analysis

RNAseq fastq files of samples were aligned to the human genome GRCh38 using HISAT2 v2.1.0. Number of reads was assigned to genes by using featureCounts v1.6.1. Reads mapped to genes were normalized using count per million method (CPM) implemented in edgeR package in R Bioconductor. To analyze genes that have the highest fold change upon different durations of stimulation with aIg (60, and 180 min), genes were ranked by the difference between their CPM normalized values and mean of CPM normalized values of this gene in the unstimulated control sample.

From FASTQ files to count tables

After sequencing, sequence data from the NextSeq500 (Illumina) was demultiplexed. Two separate FASTQ files were generated for the mRNA and protein libraries. The quality of the sequencing data was evaluated using a FastQC tool (version 0.11.7, Babraham Bioinformatics). The Python script presented by Adrian Veres (https://github.com/indrops/indrops) was used with some modifications, (in particular, for the protein libraries analysis), to process the FASTQ files in order to generate an mRNA and a protein count table. Briefly, the FASTQ files were first filtered to verify that the reads had the correct structure, sufficient quality and complexity. In our setup, 70-80% of the reads were successfully passing the filtering process. Secondly, "real" cells were determined by identifying the cell barcodes having the highest number of reads and setting a threshold below which the barcodes with fewer reads are removed (i.e., cell encapsulated with two beads, a cluster of cells encapsulated with one bead, cells with degraded mRNA). Thirdly, reads were sorted according to their barcode of origin. Subsequently, the remaining reads were aligned using Bowtie with different settings for the mRNA and protein files. For the mRNA m = 10, n = 1, l = 15, e = 1000 as described by Adrian Veres and for the proteins m = 1, n = 1, l = 8, e = 75. With m = the maximum number of different alignments allowed per read, n = the number of mismatches allowed in the first l bases of the read and e = the maximum sum allowed of the quality values al all mismatched positions. The mRNA reads were aligned to the Homo Sapiens Genome FASTA file, while the protein reads were aligned to a self-written FASTA file in which were present the 8 bp Ab tag sequences. Finally, the reads were quantified to UMIFM (UMI-filtered mapped) counts and were aggregated in two separate count tables for the mRNA and for the proteins.

Quality control, filtering and normalization

To obtain a dataset with high-quality cells several quality checks were performed. First, a Seurat (Stuart et al., 2019) data object was created, keeping cells with at least 100 genes detected, including genes that were detected in at least 100 cells across the full data-set. Cells with matching protein measurements were added as a second modality, filtering out cells that did appear in only one of the two modalities. Then, cells were filtered out with more than 4000 RNA UMIFM counts and/or less than 150 genes detected, and with more than 15% mitochondrial counts. In addition, we filtered out cells with more than 3000 protein counts and/or less than 45 pro-teins detected. The final dataset used for the further analysis, contained 6952 cells, divided over 8 samples: 648 cells at t= 0 minutes, 923 cells at t= 2 minutes, 508 cells at t= 4 minutes, 713 cells at t= 6 minutes, 863 cells at t= 60 minutes, 1099 cells at t= 180 minutes, 943 cells at t= 6 minutes + Ibrutinib, and 1255 cells at t= 180 minutes + Ibrutinib. Next, RNA counts were normalized using 'single-cell transform' implemented in the Seurat package (Hafemeister and Satija, 2019), and a scaled dataset was computed where variance in percentage of mitochondrial counts and total UMIFM counts was regressed out. Protein counts were normalized using the CLR method implemented in the Seurat package (Stuart et al., 2019), and a scaled dataset was computed where variance in number of proteins detected, total UMIFM counts and percentage of Histone H3 counts was regressed out.

QUANTIFICATION AND STATISTICAL ANALYSIS

Multi-omics Factor Analysis + (MOFA+) Model on time series of aIg stimulation

Two views were used to train a MOFA (as implemented in MOFA+, (Argelaguet et al., 2020)) including cells from time-points 0, 2, 4, 6, 60 and 180 minutes with aIg: RNA view included (single-cell transform-(Hafemeister and Satija, 2019)) normalized and scaled counts of 2159 variable genes (determined by mean.var.plot as implemented in Seurat) and the protein view included normalized (centered log ratio transformation) and scaled counts from all 80 measured (phospho)proteins. All original code to perform the filtering, detailed settings of normalization and variable gene selection, documentation of analysis and generating the figures in this study has been deposited at github and archived on zenodo (https://zenodo.org/record/5082775 https://doi.org/10.5281/zenodo.5082775). A read-able html documentation was generated using worflowR (Blischak et al., 2019) and can be accessed via GitHub Pages (https://vanbuggenum.github.io/QuRIE-seq_manuscript/).

MOFA+ default settings were used, resulting in 9 factors. A UMAP was computed on factors 1 to 7. Visualization of the B-cell signaling pathway activity (Figure 2C) with Factor 1 loadings as color scale was done using Cytoscape (Shannon et al., 2003). Enrichment analysis (using EnrichGO from clusterprofiler (Yu et al., 2012)) was used to interpret positive gene loadings of factor 3 (Figure 2F).

Multi-omics Factor Analysis + (MOFA+) model on timepoints including Ibrutinib data

A MOFA model was built using timepoints 0, 6 and 180 minutes aIg stimulated cells together with 6 and 180 minutes aIg stimulated cells in presence of Ibrutinib. The data was processed and normalized in a similar manner to the described time-series analysis. The RNA view included normalized and scaled counts of 2263 variable genes across these samples, and the protein view included normalized and scaled counts from all 80 measured (phospho)proteins. MOFA+ default settings were used, resulting in 8 factors. A UMAP was built on these factors. Loadings (gene and protein dataset) were used to interpret the factors.

Cell Reports Methods, Volume *1*

Supplemental information

Single-cell intracellular epitope and transcript detection reveals signal transduction dynamics

Francesca Rivello, Erik van Buijtenen, Kinga Matuła, Jessie A.G.L. van Buggenum, Paul Vink, Hans van Eenennaam, Klaas W. Mulder, and Wilhelm T.S. Huck

Supplementary Information

Supplementary Figures

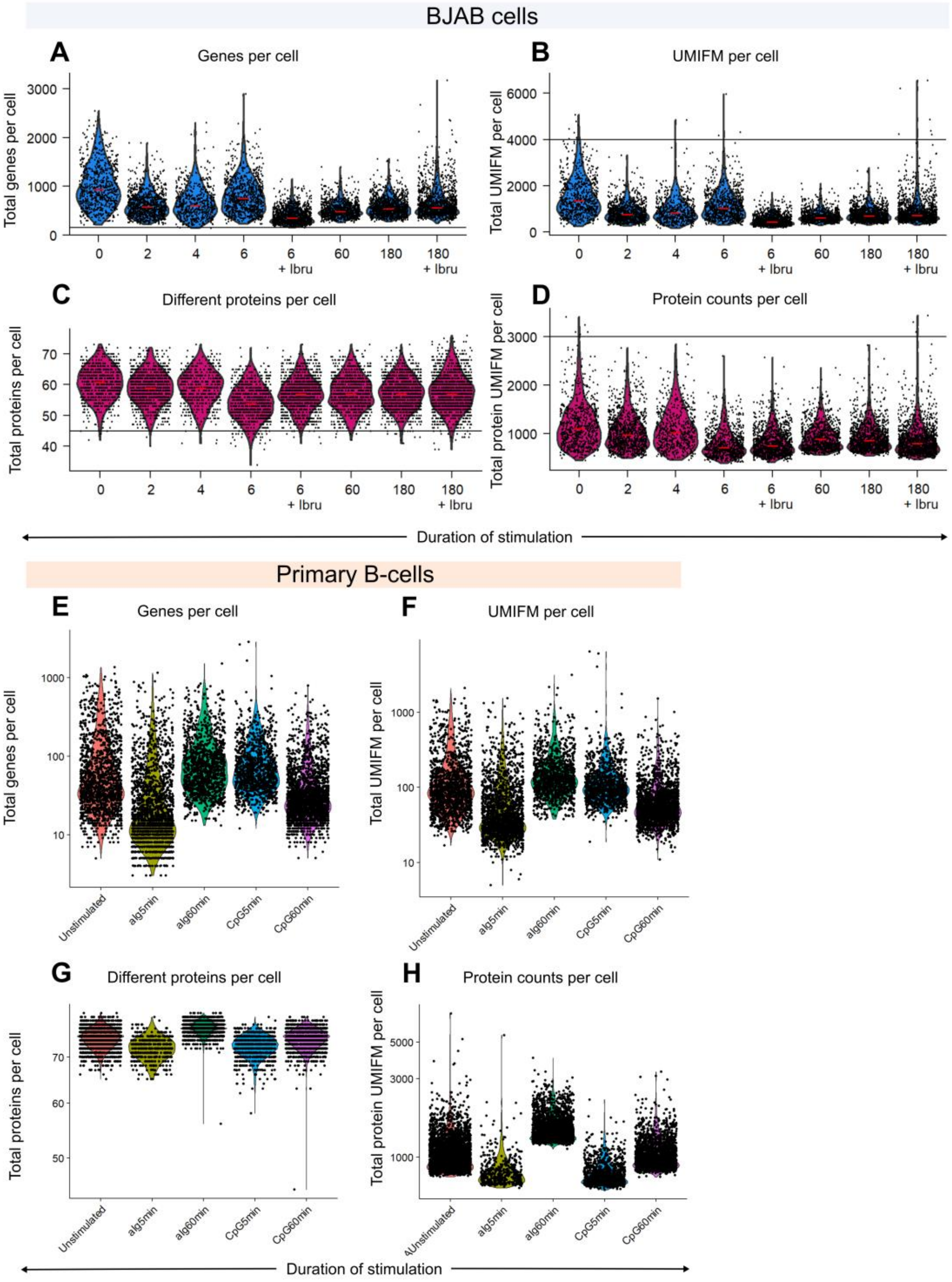

Supplementary Figure 1: Technical quality of the QuRIE-seq transcriptomic and proteomic libraries on BJAB cells (A-D) and primary B-cells isolated from PBMCs (E-H). (**A-D**) BJAB cells at eleven different stimulation and/or inhibition conditions: after stimulation with aIg for: 0, 2, 4, 6, 60 and 180 minutes, with or without prior Ibrutinib inhibition. (**E-H**) Primary B-cells isolated from PBMCs after stimulation with aIg or CpG for: 0, 5 and 60 minutes. Violin plots for (**A, B, E, F**) the transcriptomic and (**C, D, G, H**) the proteomic libraries. (**A**, **E**) The number of different genes detected per cell. (**B, F**) The number of unique molecular identifiers filtered mapped (UMIFM) detected per cell. (**C, G**) The number of different proteins detected per cell. (**D, H**) The number of protein counts per cell.

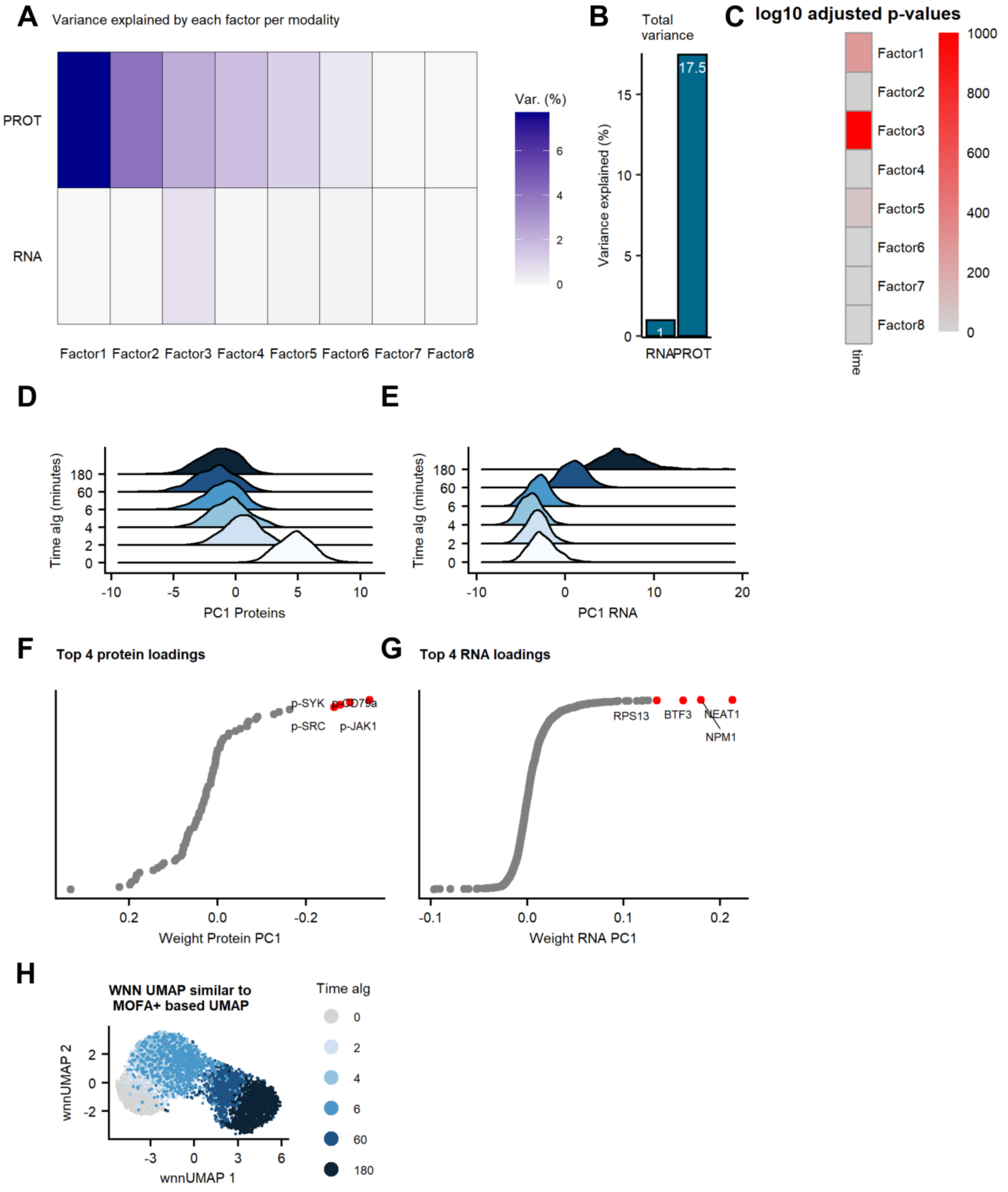

Supplementary Figure 2: Supplementary results on MOFA+ analysis related to Figure 2. (**A**) Percentage of variance explained by each factor per modality (proteins and RNA). (**B**) Percentage of the total variance explained per modality. (**C**) Pearson's correlation (p-value) between the MOFA+ factors and the duration of anti-immunoglobulin antibody (aIg) stimulation. (**D-H**) Principal component analysis (PCA) and (Weighted Nearest Neighbor Analysis) WNN analysis using the Seurat package shows similar results to

factor 1 and factor 3 determined by MOFA+ analysis. (**D**) Density plot of protein-based PC1 values as the function of time after stimulation with aIg. (**E**) Density plot of RNA-based PC1 values as function of time after stimulation with aIg. (**F**) Protein loadings contributing to PC1. The top 4 protein loadings are annotated. (**G**) RNA loadings contributing to PC1. The top 4 RNA loadings are annotated. The results are equivalent to factors 1 and 3 in MOFA+ analysis, respectively. (**H**) UMAP of weighted nearest neighbors computed from RNA and Protein PC's (first 7 dimensions) using Seurat V4.

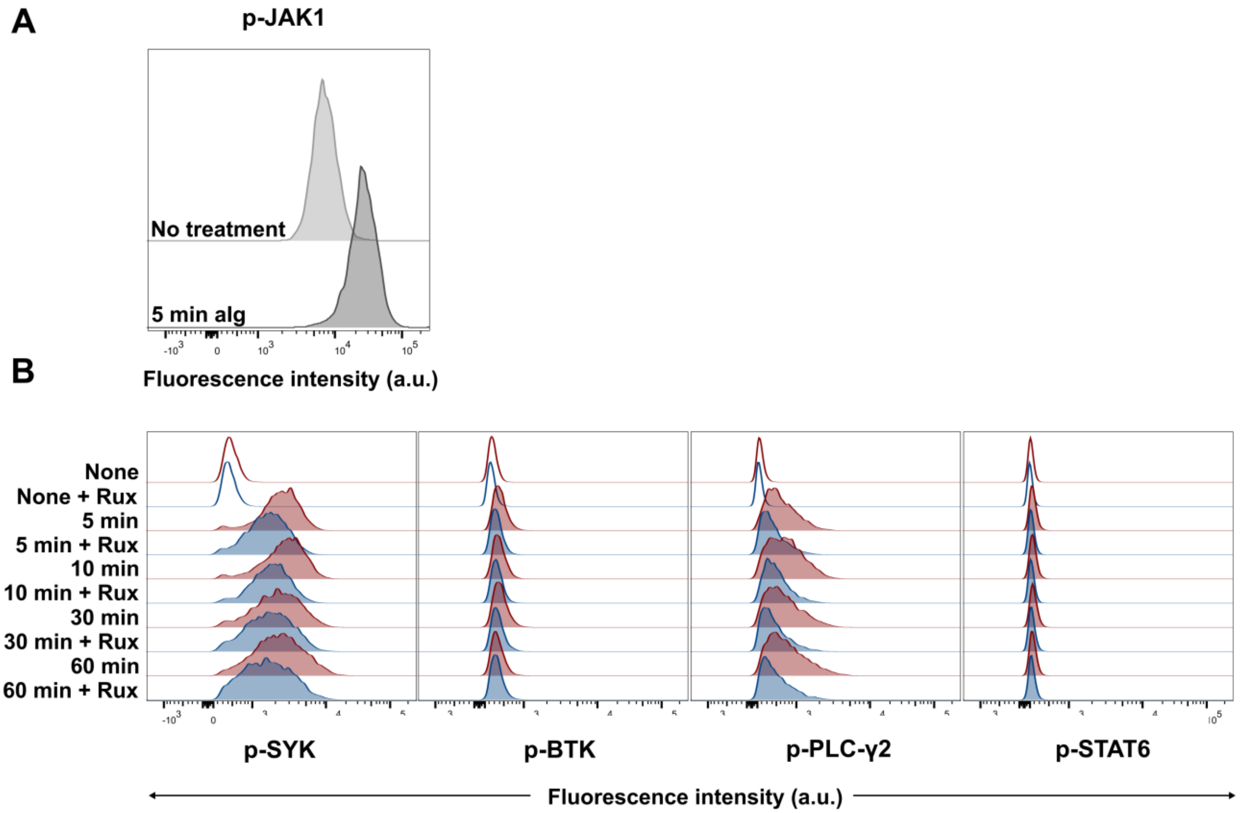

Supplementary Figure 3: The role of JAK1 in the BCR response to stimulation with aIg. Related to Figure 2. (**A**) JAK1 phosphorylation after 5 minutes of stimulation with aIg characterized by flow cytometry. (**B**) The phosphorylation level of four proteins: SYK, BTK, PLC-γ2, and STAT6 determined using flow cytometry for unstimulated BJAB cells, and stimulated with aIg for: 5, 10, 30, and 60 minutes with (blue curves) or without (red curves) prior Ruxolitinib (Rux) inhibition. *Rux is a JAK1/2 inhibitor.*

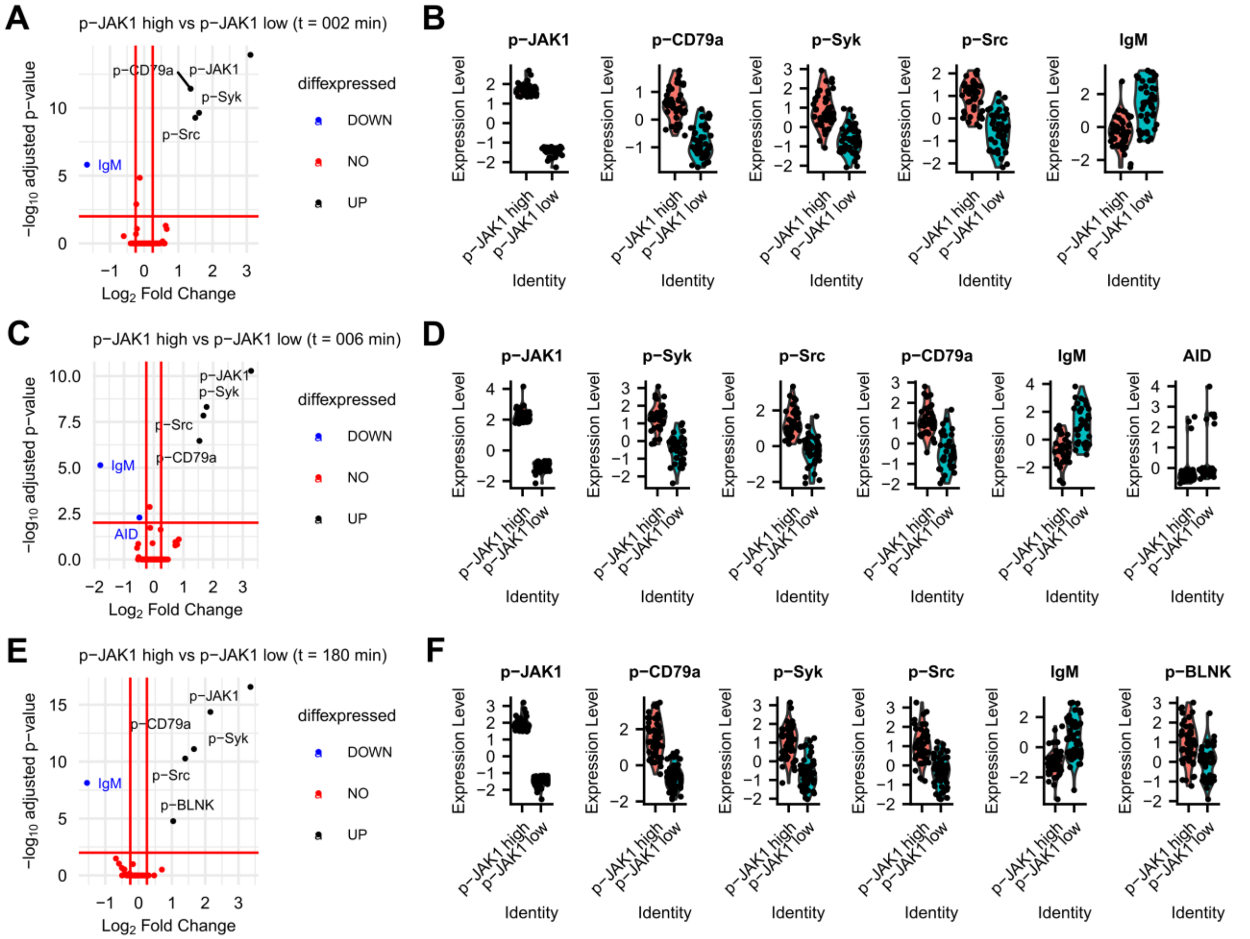

Supplementary Figure 4. Differential (phospho-)proteins of top 5% versus bottom 5% p-JAK1 cells. At timepoint t = 2 min (**A, B**), t = 6 min (**C, D**) or t = 180 min (**E, F**) differential expression analysis was performed on normalized and scaled counts, between the subset of cells with high (top 5%) p-JAK1 signal versus low (bottom 5%) p-JAK1 signal (Wilcoxon Rank Sum test). Volcano plots (**A, C, E**) show significant increased or decreased phospho-proteins (Log_2 Fold Change > 0.25 or < -0.25; adjusted p-val < 0.05). Violin plots (**B, D, F**) illustrate the highlighted significant features.

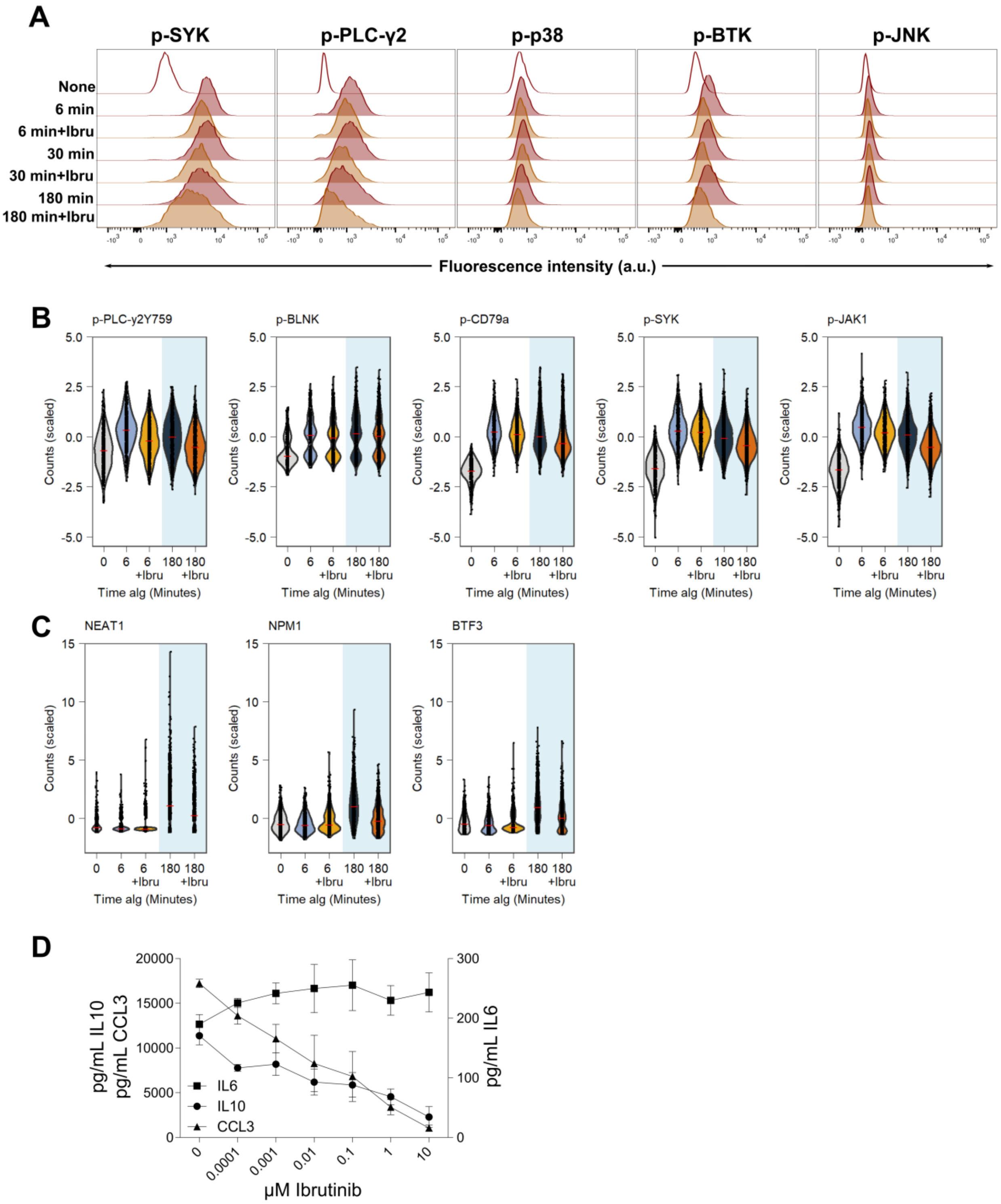

Supplementary Figure 5: Cellular response to anti-immunoglobulin antibody (aIg) stimulation in the presence of Ibrutinib (Ibru) inhibitor of Bruton's tyrosine kinase (BTK) . Related to Figure 3. (**A**) Flow cytometry characterization of the phosphorylation of SYK, PLC-γ2, p38, BTK, and JNK upon different stimulation conditions: unstimulated cells (0 minutes - red empty line); stimulation with aIg for: 6, 30, and 180 minutes with (orange filled line) and without (red filled line) prior Ibru inhibition. (**B**) QuRIE-seq

measurement of phosphoproteins. Scaled counts of signaling components up- or downstream of BTK: PLC-y2, BLNK, CD79a, SYK, and JAK1 as a function of stimulation with aIg for: 0, 6, and 180 minutes with and without Ibru. (**C**) Top 3 genes of factor 3 loadings in the QuRIE-seq measurement: *NEAT1*, *NPM1*, and *BTF3*, scaled counts as a function of stimulation with aIg for: 0, 6, and 180 minutes with and without Ibru. (**D**) ELISA analysis of BJABs culture supernatant for cytokines upon overnight (16 hours) stimulation in the presence of Ibrutinib. Ibrutinib was used to block BTK (part of BCR signaling pathway), and titrated while cells were stimulated with aIg (final concentration of 10 µg/ml). The dose-dependent decrease of cytokine secretion was observed for IL10 and CCL3, but not for IL6.

Supplementary Tables

Supplementary Table 1: Antibodies used for immunostaining of cells prior to microfluidic encapsulation. Related to STAR Methods section.

	Target	Ab barcode sequence	Vendor	Clone	Used concentration
1	**Histone H3**	**CAATCCCT**	Cell Signaling 4499BF	D1H2	0.1 µg/ml
2	**p-p65**	**GTCCAGGC**	Cell Signaling 3033BF	93H1	1 µg/ml
3	**p-SYK**	**TGTGTATA**	Cell Signaling 2710BF	C87C1	1 µg/ml
4	**p-JNK**	**AGGATCGA**	Cell Signaling 9255BF	G9	1 µg/ml
5	**p-p38**	**CACGATTC**	Cell Signaling 4511BF	D3F9	1 µg/ml
6	**p-PLC-y2**	**GTATCGAG**	R&D Systems MAB37161	790623	1 µg/ml
7	**p-BTK**	**TCTCGACT**	Biolegend 601702	A16128B	1 µg/ml
8	**p-SHP-2**	**ACCCGCAC**	Cell Signaling 5431BFF	D66F10	1 µg/ml
9	**p-SHP-1**	**CATGCGTA**	Cell Signaling 8849BF	D11G5	1 µg/ml
10	**Cyclin E**	**GTGATAGT**	ThermoFisher 32-1600	HE12	1 µg/ml
11	**c-MYC**	**TGATATCG**	ThermoFisher 700648	27H46L35	1 µg/ml
12	**p-RB**	**AGGGCGTT**	ThermoFisher 701059	14H7L14	0.1 µg/ml
13	**p-c-JUN**	**CTATACGC**	ThermoFisher MA5-27760	GT653)	1 µg/ml
14	**active Caspase-3**	**GCTCGTCA**	BD Bioscience 559565	C92-605	1 µg/ml
15	**CD70**	**TACATAAG**	BD Bioscience 555833	Ki-24	1 µg/ml
16	**IgD**	**AATTGAAC**	BD Bioscience 555776	IA6-2	1 µg/ml
17	**CD86**	**CCAGTGGA**	BD Bioscience 555655	2331 (FUN-1)	1 µg/ml
18	**CD20**	**GTCCATTG**	BD Bioscience 555677	H1	0.1 µg/ml
19	**CD79a**	**TGGACCCT**	BD Bioscience 555934	HM47	0.1 µg/ml
20	**CD5**	**AGCAGTTA**	BD Bioscience 555350	UCHT2	1 µg/ml
21	**p-AKT**	**CTTGTACC**	Cell Signaling 4060BF	D9E	1 µg/ml
22	**S6**	**GAACCCGG**	Cell Signaling 2317B	54D2	1 µg/ml
23	**ERK 1/2**	**TCGTAGAT**	Cell Signaling 4696BF	L34F12	1 µg/ml
24	**p38**	**ACGCGGAA**	Cell Signaling 8690BF	D13E1	1 µg/ml
25	**AKT**	**CGCTATCC**	Cell Signaling 4685BF	11EE7	1 µg/ml
26	**SYK**	**GTTGCATG**	Cell Signaling 13198BF	D3Z1E	1 µg/ml
27	**BTK**	**TAAATCGT**	Cell Signaling 8547BF	D3H5	0.1 µg/ml
28	**p-S6**	**ATCGCCAT**	Cell Signaling 4858BF	D57.2.2E	0.1 µg/ml
29	**p-ERK 1/2**	**CATAAAGG**	Cell Signaling 5726BF	D1H6G	1 µg/ml
30	**p65**	**TCACGGTA**	Cell Signaling 8242BF	D14E12	0.1 µg/ml
31	**CD19**	**CACTCAAC**	SantaCruz sc-373897	F-3	2 µg/ml
32	**p-Histon H3**	**GCTGTGA**	Biolegend 650802	11D8	0.1 µg/ml
33	**IgM**	**TTGCGTCG**	Biolegend 314502	MHM-88	1 µg/ml
34	**Cyclin A**	**ATATGAGA**	Biolegend 644001	E23.1	0.1 µg/ml
35	**p-Histon H2A.X**	**CACCTCAG**	Biolegend 613402	2F3	0.1 µg/ml

**36**	**Cyclin B1**	**GCTACTTC**	Biolegend 647902	V152	0.1 µg/ml
**37**	**Ki-67**	**TGGGAGCT**	Biolegend 350523	Ki-67	0.1 µg/ml
**38**	**JNK**	**ATCCGGCA**	R&D Systems AF1387		1 µg/ml
**39**	**SHP-1**	**CCGTTATG**	R&D Systems MAB1878	255402	1 µg/ml
**40**	**p-SRC**	**GGTAATGT**	R&D Systems MAB2685	1246F	1 µg/ml
**41**	**p-TOR**	**TAAGCCAC**	R&D Systems MAB1665	834115	0.1 µg/ml
**42**	**GAPDH**	**ACCGAACA**	Biolegend 607902	W17079A	1 µg/ml
**43**	**BLNK**	**GTTTGTGG**	BD Biosciences 559930	2B11	1 µg/ml
**44**	**p-BLNK**	**TAGACGAC**	BD Biosciences 558366	J117-1278	0.1 µg/ml
**45**	**p-PKC-b1**	**ACGCTTGG**	ThermoFisher 702430	3H8L1	0.1 µg/ml
**46**	**CD53**	**CGCTACAT**	BD Biosciences 555506	HI29	0.1 µg/ml
**47**	**CD38**	**GAAAGACA**	Biolegend 303535	HIT2	0.1 µg/ml
**48**	**CD45**	**TTTGCGTC**	Biolegend 304045	HI30	0.1 µg/ml
**49**	**p-IRAK4**	**ATGGTCGC**	Cell Signaling 11927S	D6D7	1 µg/ml
**50**	**p-CD79a**	**CGACATAG**	Cell Signaling 14732BF	D1B9	0.1 µg/ml
**51**	**p-CDK1**	**GATTCGCT**	ThermoFisher 701808	17H29L7	1 µg/ml
**52**	**p-CDK4**	**TCCAGATA**	ThermoFisher 702556	9H2L7	1 µg/ml
**53**	**p-AMPK-a1/2**	**ACTACTGT**	ThermoFisher701068	10H2L20	0.1 µg/ml
**54**	**p-AMPK-b1**	**CGGGAACG**	Thermo Fisher 700241	9H26L42	0.1 µg/ml
**55**	**T-bet**	**GACCTCTC**	Biolegend 644825	4B10	1 µg/ml
**56**	**p-IKK a/b**	**TTATGGAA**	ThermoFisher 701643	7H17L17	1 µg/ml
**57**	**CD27**	**ACAGCAAC**	Abcam ab192336	EPR8569	0.1 µg/ml
**58**	**p-JAK1**	**CGCAATTT**	ThermoFisher 700028	59H4L5	1 µg/ml
**59**	**p-PLC-y2 (Y759)**	**GAGTTGCG**	R&D Systems MAB7377	744757	0.1 µg/ml
**60**	**IL10**	**TTTCGCGA**	ThermoFisher 16-7108-85	JES3-9D7	1 µg/ml
**61**	**CCL3/4**	**ACCAGTCC**	R&D Systems MAB2701-100	93342	1 µg/ml
**62**	**CD80**	**CTTTCCTT**	Biolegend 305212	2D10	1 µg/ml
**63**	**p-CDK6**	**CTGGACGT**	ThermoFisher	16HCLC	1 µg/ml
**64**	**p-STAT1**	**GAACGGTC**	ThermoFisher 33-3400	ST1P-11A5	1 µg/ml
**65**	**p-STAT3**	**TGTTCACG**	Biolegend 690402	A16002B	1 µg/ml
**66**	**p-STAT5**	**ATCTGATC**	ThermoFisher 701063	6H5L15	1 µg/ml
**67**	**p-STAT6**	**GAGAAGGG**	ThermoFisher 700247	46H1L12	1 µg/ml
**68**	**KLF6**	**TCACTCCT**	ThermoFisher 39-6900	9A2	1 µg/ml
**69**	**BCL6**	**AGATAACA**	BD Bioscneces 561520	K112-91	1 µg/ml
**70**	**AID**	**CTTATTTG**	BD Biosciences 565784	EK2-5G9	1 µg/ml
**71**	**IgG**	**GCGGGCAT**	BD Biosciences 555784	G18-145	0.1 µg/ml
**72**	**CD24**	**TACCCGGC**	RnD Systems MAB5247	ML5	1 µg/ml
**73**	**IgA**	**ATTGTTTC**	Biolegend 411502	HP6123	0.5 µg/ml
**74**	**IgE**	**CGCAGGAG**	Biolegend 325502	MHE-18	1 µg/ml
**75**	**CD23**	**GCACCAGT**	Abcam ab245732	SP163	0.1 µg/ml

**76**	**BLIMP1**	**TAGTACCA**	RnD Systems MAB36081	646702	1 µg/ml
**77**	**IRF8**	**ATTCGTGC**	Biolegend 656502	656502	1 µg/ml
**78**	**IRF4**	**CGCGTGCA**	ThermoFisher 14-9858-82	3 E 4	1 µg/ml
**79**	**BAFF-R**	**GAATACTG**	RnD Systems MAB1162	2403C	1 µg/ml
**80**	**XBP1**	**TCGACAAT**	Abcam ab239954	EPR4086	1 µg/m l

Supplementary Table 2: Antibodies used for flow cytometry analysis of BJABs. Related to STAR Methods section.

Target	Fluorophore	Vendor	Clone	Used concentration
**p-SYK**	PE	Cell Signaling 6485	C87C1	1/50
**p-PLC-y2**	Alexa 647	BD Bioscience 558498	K86-689.37	1/100
**p-BTK**	PE	Biolegend 601704	A16128B	1/50
**p-p38**	Alexa 488	Cell Signaling 41768S	3D7	1/50
**p-AKT**	PE	Cell Signaling 5315	D9E	1/50
**p-ERK 1/2**	PE	Cell Signaling 75765S	D1H6G	1/50
**p-JNK**	Alexa 647	Cell Signaling 9257S	G9	1/50
**p-p65**	Alexa 647	Cell Signaling 5733	93H1	1/50
**p-S6**	PE	Cell Signaling 5316	D57.2.2E	1/800

Supplementary Table 3: Sequences of all the primers used for library preparation. Related to STAR Methods section. All primers were ordered from Biolegio (The Netherlands).

	**Primer sequence 5’ -> 3’**	**Length (bp)**
**PE2-N6**	TCGGCATTCCTGCTGAACCGCTCTTCCGATCT NNNNNN	38
**PE1**	CAAGCAGAAGACGGCATACGAGAT [6-bp library index] CTCTTTCCCTACACGA	46
**PE2**	AATGATACGGCGACCACCGAGATCTACACGGTCTCGGCATTCCTGCTG AAC	51
**Custom Read 1 primer**	GGCATTCCTGCTGAACCGCTCTTCCGATCT	30
**Custom Index Read primer**	AGATCGGAAGAGCGTCGTGTAGGGAAAGAG	30
**Custom Read 2 primer**	CTCTTTCCCTACACGACGCTCTTCCGATCT	30
